# Pathogenesis of Graves’ Disease Determined Using Single-Cell Sequencing with Thyroid Autoantigen Peptide Stimulation in B Cells

**DOI:** 10.3390/cells14141102

**Published:** 2025-07-17

**Authors:** Genki Kobayashi, Takuro Okamura, Yoshitaka Hashimoto, Kimiko Sakai, Madoka Sumi, Dan Imai, Nobuko Kitagawa, Masahide Hamaguchi, Takahiro Tsujikawa, Shigeru Hirano, Michiaki Fukui

**Affiliations:** 1 Department of Endocrinology and Metabolism, Graduate School of Medical Science, Kyoto Prefectural University of Medicine, Kyoto 602-8566, Japan; genkoba@koto.kpu-m.ac.jp (G.K.); d04sm012@koto.kpu-m.ac.jp (T.O.); k-sakai@koto.kpu-m.ac.jp (K.S.); sumi0220@koto.kpu-m.ac.jp (M.S.); dimai@koto.kpu-m.ac.jp (D.I.); nobuko-s@koto.kpu-m.ac.jp (N.K.); michiaki@koto.kpu-m.ac.jp (M.F.); 2 Department of Diabetes and Endocrinology, Matsushita Memorial Hospital, Moriguchi 570-8540, Japan; y-hashi@koto.kpu-m.ac.jp; 3 Department of Otolaryngology–Head and Neck Surgery, Graduate School of Medical Science, Kyoto Prefectural University of Medicine, Kyoto 602-8566, Japan; tu-ji@koto.kpu-m.ac.jp (T.T.); hirano@koto.kpu-m.ac.jp (S.H.)

**Keywords:** single-cell RNA sequencing, Graves’ disease, B cells, thyroid autoantigens, peptide stimulation, autoantibody production

## Abstract

This study reports the use of single-cell RNA sequencing to evaluate B cells in the peripheral blood mononuclear cells (PBMCs) and intrathyroidal blood mononuclear cells of patients with Graves’ disease (GD) undergoing thyroidectomy. These cells were stimulated with overlapping peptides of thyroid autoantigens, including thyroid-stimulating hormone receptor (TSHR), thyroglobulin (Tg), and thyroid peroxidase (TPO). In PBMCs, naive B cells are characterized by *IL6* and *CXCR5*, whereas memory B cells express *IGHG1*, *IGHG2*, and *CD74*. *HLA-DMA*, *HLA-DRB1*, *IGHG*, *IGHM*, *CD74*, *CD79A*, and *MS4A1* expression increased in peptide-stimulated naive and memory B cells compared to those in the controls. Thyroid naive B cells are characterized by *CD40* and *TNFRSF13C*, whereas memory B cells express *IGHM*, *CD79A*, and *MS4A1.* Thyroid B cells showed higher *DUSP1*, *DUSP2*, *CD69*, *FOSB*, *RGS1*, and immunoglobulin gene expression than control PBMCs and thyroid cells. B-cell receptor analysis revealed frequent *IGHV3-23* and *IGHV4-34* usage in controls, whereas *IGHV4-34/IGHJ4* expression was increased in TSHR-stimulated groups. We concluded that B-cell responses to TSHR, Tg, and TPO differed and that changes in B-cell reactivity also occurred in PBMCs and the thyroid. Additionally, *IGHV3-23* and *IGHV4-34* may be associated with autoantibody production in GD.

## 1. Introduction

Graves’ Disease (GD) is an autoimmune thyroid disorder caused by the breakdown in immune tolerance against thyroid autoantigens, involving the clinical symptoms of enlarged thyroid gland, weight loss, rapid or irregular heartbeat, tremors, and thickening and discoloration in the skin. The formation of autoantibodies stimulating the thyrotropin hormone receptor (TSHR) referred to as TRAb are the hallmark of GD and are the predominant driver of pathogenesis with Th1 cytokine inflammation and insulin growth factor-1 receptor (IGFR) signaling playing a supportive role in Graves’ orbitopathy and extra-thyroid manifestations [1,2]. The levels of TRAb directly correlate with the serum levels of thyroid hormones, T3 and T4, the disease severity scores and response to anti-thyroid drugs. The presence of anti- thyroid peroxidase (TPO) and anti-thyroglobulin (Tg) autoantibodies do not contribute to the GD pathogenesis per se but are rather representative of tissue damage and the release of these abundant autoantigens from thyroid follicles. In addition to TSHR, antibodies against Tg and TPO are also associated with thyroid autoimmunity [3]. Many patients with GD have antibodies against Tg and TPO, which are characteristic of Hashimoto’s disease [4,5]. These autoantibodies exhibit polyclonality and are thought to occur secondary to thyroid cell destruction [6]. Previous reports have indicated that T cells in patients with GD recognize Tg, TPO, and TSHR as antigens [7]. The three major thyroid autoantigens used in this study—TSHR, Tg, and TPO—have all been structurally elucidated in recent years. These structural breakthroughs, particularly for Tg and TPO, have enabled the precise design of stimulation peptides derived from known antigenic regions [8,9,10].

The three main treatment options available are antithyroid drugs, radioactive iodine therapy, and surgery. Radioactive iodine therapy cannot be used during pregnancy and carries the risk of worsening GD ophthalmopathy [1,11]. Surgical therapy presents complications because of its invasiveness and difficulty, as well as postoperative hypothyroidism [12]. Therefore, antithyroid drugs such as methimazole or propylthiouracil are often used as the initial approach. However, adverse effects such as agranulocytosis, severe liver dysfunction, and antineutrophil cytoplasmic antibody-associated vasculitis can occur [12], resulting in the risk of death; thus, developing safe drugs is urgent. Currently, there are no treatments that target the autoimmune response, which is the main cause of GD pathogenesis.

In recent years, single-cell RNA sequencing (scRNA-seq), a new technology enabling single-cell profiling using next-generation sequencers, has enabled the study of the diversity of immune cells [13]. Furthermore, in recent SARS-CoV-2 research, overlapping peptides have been designed so that each peptide overlaps by 11 amino acid residues back and forth in a peptide pool consisting of 15 mers, which has been the focus of [14,15]. These peptides efficiently stimulate T cells in an antigen-specific manner. T cells play a crucial role in B-cell activation. In patients with GD, antigens bind to B-cell receptors (BCR) and activate B cells through interactions with surface molecules such as CD40, CD80, and CD86, leading to increased TRAb production [16]. Activated B cells produce cytokines that enhance thyroid inflammation [6]. Moreover, in autoimmune thyroid diseases, receptor revision occurs in lymphoid follicles within the thyroid gland, potentially contributing to high-affinity thyroid autoantibody generation [17].

To date, no studies have evaluated B cells in the inflammatory state in GD. Therefore, we stimulated peripheral blood mononuclear cells (PBMCs) and intrathyroidal blood mononuclear cells from patients with GD using overlapping peptides of thyroid autoantigens and conducted scRNA-seq analysis, focusing specifically on B cells, to elucidate the immune status in GD and develop treatments that target the immune response.

## 2. Materials and Methods

### 2.1. Sex as a Biological Variable

This study included samples from both men and women diagnosed with GD. However, sex was not explicitly considered as a biological variable in the study design or analysis. This study focused on B-cell responses to thyroid autoantigens, and the findings are expected to be generally applicable regardless of sex.

### 2.2. Study Design and Participants

This study began on 8 November 2021, targeting patients with GD aged 16 and older who were scheduled for thyroidectomy. Serum TRAb levels were assessed at the time of initial consultation at our hospital, and a cut-off value of >2 IU/L was used to define TRAb positivity, in line with standard clinical guidelines. PBMCs were collected within one month before the surgery, and a 1 cm × 1 cm × 1 cm portion of thyroid tissue was taken during the surgery.

### 2.3. PBMC Isolation and In Vitro Culture

Peripheral blood (approximately 10 mL) was drawn into cell preparation (CPT) tubes (BD Vacutainer^®^ CPT™ Mononuclear Cell Preparation Tube, BD Biosciences, San Jose, CA, USA). Subsequently, 1.5 × 10^6^ PBMCs from each group were plated in a 96-well plate with Roswell Park Memorial Institute Medium (RPMI) (Nacalai Tesque, Kyoto, Japan) supplemented with 5% human AB serum. The control (Ctrl) group received only sterile phosphate-buffered saline (PBS, pH 7.4), while TSHR overlapping peptides were added to the TSHR group, Tg-overlapping peptides to the Tg group, and TPO-overlapping peptides to the TPO group at a concentration of 0.3 nmol/mL. The plates were incubated for 2 h at 37 °C in a CO_2_ incubator. The overlapping peptides were chemically synthesized by GL Biochem, Ltd. (Shanghai, China). The sequences are listed in Supplementary Table S1. Each peptide powder (purity ≥ 70–75%) was dissolved in dimethyl sulfoxide (DMSO) to obtain a final concentration of 100 mg/mL. Specifically, 1 mg of each peptide was sequentially dissolved in 0.98 mL of DMSO to create a pooled solution. For example, 98 peptides of thyroglobulin origin were each dissolved one-by-one by transferring the DMSO solution from one tube to the next, ensuring complete dissolution of each peptide into a single 0.98 mL pooled solution. Similarly, pooled peptide mixtures of LTSHR and TPO origin were also prepared by dissolving their respective peptide sets in appropriate volumes of DMSO (2.31 mL for 231 mg, and 1.77 mL for 177 mg, respectively). The resulting stock solution (100 mg/mL) was then aliquoted into 1.5 mL tubes at 100 μL per tube and stored at −30 °C. For experimental use, a working solution was prepared by diluting 0.15 μL of this stock into 150 μL of sterile pure water, yielding a final peptide concentration of 100 μg/mL. The pure water used for dilution was freshly opened to ensure sterility. Although no additional sterilization step such as filtration was performed after dilution, all solutions were handled aseptically, and the final aliquots for biological experiments were stored at −30 °C until use.

### 2.4. Isolation of Intrathyroidal Blood Mononuclear Cells and In Vitro Culture

A portion of the surgically excised thyroid tissue was extracted. The samples were stored in cold RPMI medium supplemented with 2% fetal bovine serum (FBS) (Gibco™, Thermo Fisher Scientific, Waltham, MA, USA) until use. The thyroid tissue was placed on a cell strainer, and after finely chopping the thyroid tissue, RPMI + 2% FBS was added multiple times. The suspension was then centrifuged at 500× *g* for 4 min at 22 °C, and the supernatant was discarded. The cell pellet was resuspended in 5 mL of 40% Percoll^®^ (Cytiva, Marlborough, MA, USA) and carefully layered on top of 5 mL of 60% Percoll^®^ in a centrifuge tube. Density gradient centrifugation was performed at 1500× *g* for 10 min at 22 °C. Blood mononuclear cells from the middle layer were gently extracted using a 1 mL pipette. Extracted blood mononuclear cells were treated with RBC lysis buffer (Sigma-Aldrich, St. Louis, MO, USA) and washed twice with 2% FBS/PBS (pH 7.4). Subsequently, the cells were stimulated with TSHR-, Tg-, and TPO-overlapping peptides in a 96-well plate, following the same procedure used for PBMCs.

### 2.5. Single-Cell Sequencing and Data Analysis

The cells were labeled with a specific sample tag (BD Rhapsody Human Single-Cell Multiplexing Kit) and stained with AbSeq antibodies against key human immune markers (BD AbSeq Immune Discovery Panel, 30 markers) for 30 min on ice. After thorough washing, equal volumes of cells with different sample tags were combined, and up to 20,000 cells were loaded into a BD Rhapsody Cartridge. Single-cell capture and library preparation were performed using the BD Rhapsody Express Single-Cell Analysis System (BD Biosciences) and the BD Rhapsody Targeted mRNA and AbSeq Reagent Kit, respectively, according to the manufacturer’s protocol. Briefly, cells were captured on beads within a microwell plate after loading, followed by cell lysis, bead retrieval, cDNA synthesis, and template switching. Library preparation for the targeted genes (BD Rhapsody Immune Response Panel Hs, 399 genes), BCR gene segments, BD AbSeq 30 markers, and multiple sample tags were performed separately according to the manufacturer’s instructions, after which the libraries were pooled before sequencing. Sequencing was performed on Illumina HiSeq X and NovaSeq X Plus platforms (Illumina, San Diego, CA, USA).

Sequencing-derived FASTQ files were processed using the BD Rhapsody Targeted Analysis Pipeline with V(D)J processing (BD Biosciences) on the Seven Bridges Platform (https://www.sevenbridges.com/d (accessed on 1 May 2024)). Initially, low-quality read pairs were filtered based on factors such as read length, average base quality score, and single-base frequency. High-quality R1 reads were used to identify cell labels and unique molecular identifiers (UMIs). High-quality R2 reads were aligned to the reference sequences, including 399 targeted genes, BD AbSeq 30 markers, and BCR gene segments, using the Bowtie2 program and the BCR gene data from the International ImMunoGeneTics Information System (IMGT.org). IGBLAST was used to identify the CDR3 regions. The reads with matching cell labels, UMI sequences, and genes were collapsed into single molecules. The resulting RDS files from the Seven Bridges Platform were imported into the R version 4.4.1 (R Foundation for Statistical Computing, Vienna, Austria). PBMC and intrathyroidal blood mononuclear cell files were separately integrated. QC was performed to exclude cells that were significantly smaller and had fewer expressed genes (“dead cells”). In this study, the BD gene panel was used, and applying the condition of nFeature_RNA < 200 left almost no cells. Therefore, QC was performed under the following conditions: 20 < nFeature_RNA < 2000 and 200 < nCount_RNA < 25,000.

After that, batch effect correction was applied, followed by dimensionality reduction and clustering using the ‘Seurat’ package in R.

### 2.6. Proteomic Analysis

After 2 h of in vitro culture with overlapping peptides, 50 μL of the supernatant from the added RPMI was collected, and protein profiling was performed using O-link technology. The O-link^®^ target 96 inflammation (Aproscience, Tokushima, Japan) was used to evaluate cytokines related to inflammation.

### 2.7. BCR Heavy and Light Chain Pairing and Frequency Distribution

BCR heavy and light chain pairing analysis was performed on naive B, memory B, and total B (defined as the combination of naive and memory B cells) cells for each of the Ctrl, TSHR, Tg, and TPO groups. Dominant BCR heavy chain V and J genes and light chain V and J gene segments were selected, and pairs with valid V-J gene annotations were counted. The frequency of each pair was calculated as a percentage of the total number of heavy and light chain pairs in each sample. Bubble plots were generated to visualize the results, with the bubble sizes representing the frequency of each pair.

### 2.8. BCR CDR3 Sequence Identification

The sequence logos were generated using the ‘ggseqlogo’ package in R, and they were used to visualize amino acid sequences.

### 2.9. Statistical Analysis

Data analysis and figure generation were performed using R and Adobe Acrobat (version 2024.003.20054; Adobe Inc., San Jose, CA, USA). For comparisons across the four groups, a one-way analysis of variance (ANOVA) followed by Tukey’s HSD post hoc test was applied. The Wilcoxon rank-sum test was used to compare two groups that did not follow a normal distribution. Fisher’s exact test was used to assess the relationships between categorical variables. A *p*-value of less than 0.05 was considered statistically significant.

## 3. Results

### 3.1. Participants’ Background

The participant profiles are presented in Table 1. Blood and thyroid samples were collected from eight individuals aged 17–58 years (two males and six females). Due to the number of cells, the final analysis by scRNA-seq included PBMCs from eight control (Ctrl) samples, five samples stimulated with overlapping peptides, intrathyroidal blood mononuclear blood cells from seven Ctrl samples, and one sample stimulated with overlapping peptides. Reasons for thyroidectomy included the adverse effects of antithyroid drugs, difficulty in treatment, and thyroid enlargement. After quality control (QC), the final dataset comprised 38,799 PBMCs (2711 cells in the TSHR group, 3920 in the Tg group, 6318 in the TPO group, and 25,850 in the Ctrl group) and 30,558 intrathyroidal blood mononuclear blood cells (89 cells in the TSHR group, 118 in the Tg group, 147 in the TPO group, and 30,204 in the Ctrl group).

### 3.2. Single-Cell RNA Sequencing of PBMCs in Patients with GD After Administration of Overlapping Peptides

**Figure 1:**
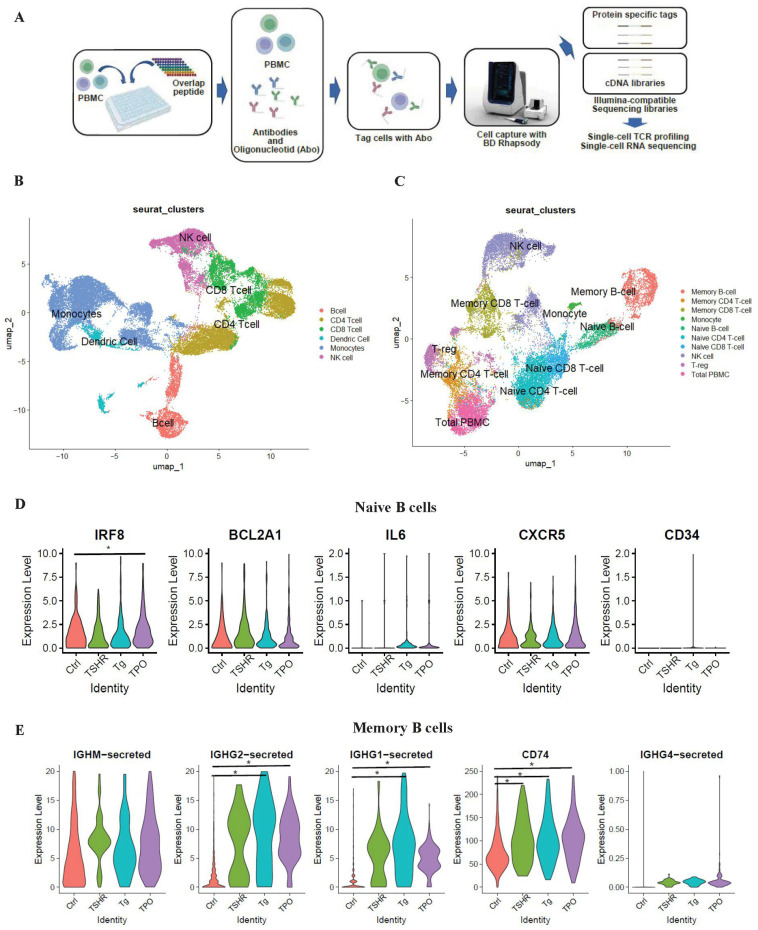
Cluster identification of PBMCs. (**A**) Experimental study design framework. (**B**) Cluster identification of PBMCs by manual reference to the genes of immune cells in the Human Protein Atlas. (**C**) Cluster identification of lymphoid cells in PBMCs by manual reference to the genes of immune cells in the Human Protein Atlas. (**D**) Violin plots of the top 5 marker genes in naive B-cell clusters in PBMCs by antigen. (**E**) Violin plots of the top 5 marker genes in memory B-cell clusters in PBMCs by antigen. Data were analyzed using one-way ANOVA with Tukey’s honest significant difference test. *: *p* < 0.05. PBMC: peripheral blood mononuclear cell; TSHR: thyroid-stimulating hormone receptor; Tg, thyroglobulin; TPO, thyroid peroxidase.

Immune cell stimulation in peripheral blood by overlapping peptides of thyroid autoantigens was used to simulate the inflammatory state of GD, followed by scRNA-seq (Figure 1A). All cells were classified into 20 major clusters based on Uniform Manifold Approximation and Projection (UMAP) dimensionality reduction. Using the “FindAllMarkers” package in R, genes characteristically expressed in each cluster were evaluated. Based on the Human Protein Atlas (https://www.proteinatlas.org/humanproteome/immune+cell (accessed on 15 May 2024)), cells were classified into B, CD4 T, CD8 T, dendritic, and NK cells, as well as monocytes (Figure 1B). Furthermore, only lymphocytes (NK, T, and B cells) were extracted, and dimensionality reduction by UMAP was performed again, resulting in 15 major clusters. Cells were then classified into immune cell types based on the genes characteristically expressed in each cluster (Figure 1C). Clustering was also performed for each overlapping peptide used for stimulation (Supplementary Figure S1A,B).

The top five characteristic genes of each cluster in Figure 1C are shown in a dot plot (Supplementary Figure S1C), and the top five genes of the naive and memory B-cell clusters are shown in violin plots for the Ctrl, TSHR, Tg, and TPO groups (Figure 1D,E). In naive B cells, genes related to immune cell activation and inflammation, such as IRF8, IL6, and CXCR5, were observed, with IRF8 showing a significant difference between the Ctrl and TPO groups. Memory B cells were characterized by the expression of genes related to immunoglobulins, notably IGHG1 and IGHG2. We evaluated cytokine-related genes in both naive and memory B cells, and we additionally assessed TNFRSF17 expression specifically in memory B cells (Supplementary Figure S1D–F). In naive B-cell clusters, IL32 and TNF expressions were increased by overlapping peptide stimulation.

### 3.3. Gene Expression Changes Induced by Overlapping Peptide Stimulation

**Figure 2:**
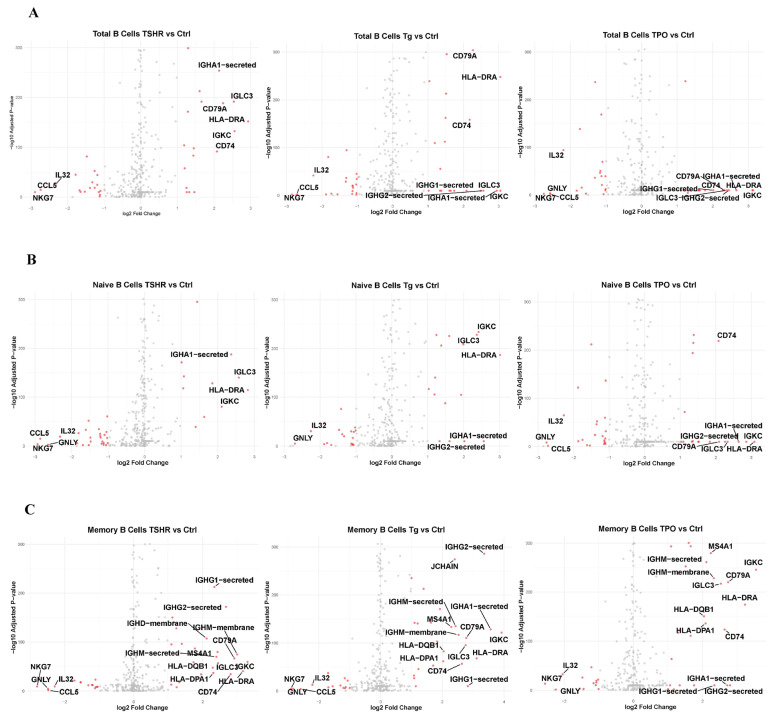
Gene expression in B-cell clusters of PBMCs. (**A**) Volcano plot for each antigen in total B cells compared to that in the control group. (**B**) Volcano plot for each antigen in naive B cells compared to that in the control group. (**C**) Volcano plot for each antigen in memory B cells compared to that in the control group. Data were analyzed using the Wilcoxon rank-sum test. PBMC: peripheral blood mononuclear cell; TSHR: thyroid-stimulating hormone receptor; Tg, thyroglobulin; TPO, thyroid peroxidase.

The changes in gene expression in the TSHR, Tg, and TPO groups compared to those in the control group are shown as volcano plots for three subsets of the PBMC group as follows: naive B, memory B, and total B cells (Figure 2A–C). In Figure 2A–C, gene names are displayed for those with an absolute log2 fold change (log2FC) of 2 or greater. We then extracted genes with an absolute value of log2FC of 1 or more and listed them in Supplementary Table S2A–C. In all subsets, the expression of genes belonging to MHC class II, such as *HLA-DMA* and *HLA-DRB1*, and genes related to immunoglobulins, such as *IGHG* and *IGHM*, increased. Additionally, genes such as *CD74*, which binds to MHC class II and is involved in antigen presentation; *CD79A*, a part of the BCR involved in signal transduction after antigen recognition; and *MS4A1*, which encodes *CD20* related to B-cell differentiation and activation, also showed increased expression. In contrast, genes involved in cytotoxicity that are typically expressed in NK cells and cytotoxic T cells, such as *GNLY*, *GZMA*, *GZMB*, and *GZMH*, exhibited decreased expression.

### 3.4. Single-Cell RNA Sequencing of Intrathyroidal Blood Mononuclear Cells in Patients with GD After Administration of Overlapping Peptides

**Figure 3:**
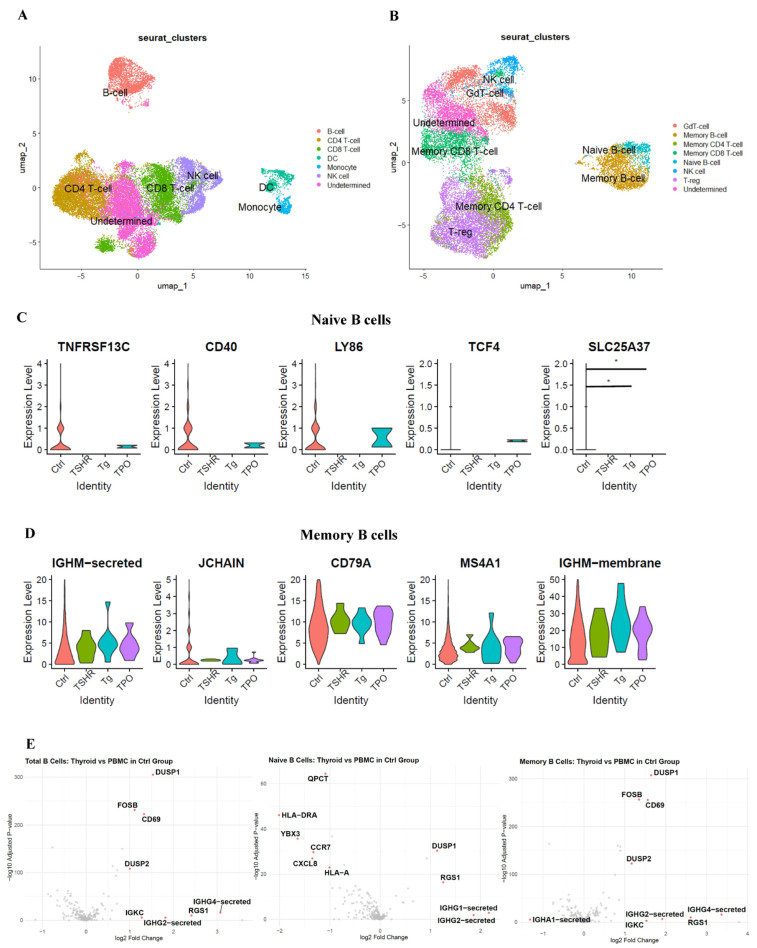
Cluster identification of intrathyroidal blood mononuclear cells and gene expression in B-cell clusters. (**A**) Cluster identification of intrathyroidal blood mononuclear cells by manual reference to the genes of immune cells in the Human Protein Atlas. (**B**) Cluster identification of intrathyroidal lymphoid cells by manual reference to the genes of immune cells in the Human Protein Atlas. (**C**) Violin plots of top 5 marker genes in intrathyroidal naive B-cell clusters by antigen. (**D**) Violin plots of top 5 marker genes in intrathyroidal memory B-cell clusters by antigen. (**E**) Volcano plot showing differential gene expression of each B-cell subtype between thyroid and PBMC control groups. Data were analyzed using one-way ANOVA with Tukey’s honest significant difference test and the Wilcoxon rank-sum test. *: *p* < 0.05. PBMC: peripheral blood mononuclear cell; TSHR: thyroid-stimulating hormone receptor; Tg, thyroglobulin; TPO, thyroid peroxidase.

Intrathyroidal blood mononuclear cells were stimulated with overlapping peptides of thyroid autoantigens, and scRNA-seq was performed. The cells were classified into 14 major clusters. Similar to the PBMC analysis, cells were classified into each immune cell type based on the genes characteristically expressed in each cluster, referring to the Human Protein Atlas (Figure 3A). Additionally, only lymphocytes were extracted and dimensionality reduction using UMAP was repeated (Figure 3B). Clustering was also performed for each overlapping peptide used for stimulation (Supplementary Figure S2A,B). The top five genes characteristically expressed in each cluster in Figure 3B are displayed in a dot plot (Supplementary Figure S2C), and the top five genes in the naive and memory B-cell clusters are shown in violin plots (Figure 3C,D). In naive B cells, genes related to B-cell maturation, such as *CD40*, *TNFRSF13C*, and *TCF4*, were observed. Memory B cells were characterized by gene expression, such as *IGHM*, and those related to B-cell activation, such as *CD79A* and *MS4A1*.

### 3.5. Evaluation of Gene Expression Changes Between the Ctrl Group of Intrathyroidal Blood Mononuclear Cells and the Ctrl Group of PBMCs

Changes in gene expression in the Ctrl group of intrathyroidal blood mononuclear cells compared to those in the Ctrl group of PBMCs are shown in Figure 3E. Genes involved in signal transduction, B-cell activation, antibody production, and the regulation of immune responses, such as *DUSP1*, *DUSP2*, *CD69*, *FOSB*, *RGS1*, and immunoglobulin, showed significantly increased expression in the thyroid group compared to that in the controls.

### 3.6. Proteomic Analysis Following Overlapping Peptide Stimulation

Box plots are displayed for proteins in the supernatant that showed significant changes after stimulation with the overlapping peptides in PBMCs and intrathyroidal mononuclear cells (Supplementary Figure S3). An increase in the concentration of several inflammation-related proteins was observed, and the number of proteins showing significant changes was greater in the blood mononuclear cells of the thyroid group than in PBMCs. Notably, in the TSHR group, an increase in IL-10RB, which is a part of the IL-10 receptor and mediates immunosuppression via IL-10 and IL-7, a protein involved in B-cell development, was observed. Additionally, the upregulation of IL-18, which is involved in IFNɤ production, and TRAIL, associated with apoptosis, was also detected in the thyroid group.

### 3.7. Evaluation of BCR Heavy and Light Chain of PBMCs

Supplementary Figures S4–S6 display the combinations of BCR heavy chain V and J genes and light chain V and J genes in the control, TSHR, Tg, and TPO groups of PBMCs, shown as bubble plots for total B, naive B, and memory B cells, respectively. Table 2 shows the top five most frequent combinations of the BCR heavy chain V and J genes and light chain V and J genes in the control group for each B-cell cluster of PBMCs. Furthermore, comparisons of the frequencies of the top five combinations of BCR heavy chain V and J genes and light chain V and J genes in the TSHR, Tg, and TPO groups, compared to those in the control group for each B-cell cluster of PBMCs, are presented in Table 3A–C. In the control group, genes associated with autoimmune diseases, such as *IGHV4-34* and *IGHJ4*, as well as those linked to leukemia, including *IGHV3-23*, *IGHV3-48*, and *IGHV3-30*, were highly prevalent. The *IGHV4-34/IGHJ4* combination was significantly increased in the TSHR group of both naive and memory B cells compared to that in the Ctrl group.

### 3.8. Evaluation of the Amino Acid Sequence in the CDR3 Region of B Cells of GD

The amino acid sequences in the CDR3 regions of the BCR heavy and light chains in the Ctrl, TSHR, Tg, and TPO groups of PBMCs are displayed as sequence logo plots for total B cells, naive B cells, and memory B cells (Supplementary Figure S7A–C). Additionally, the amino acid sequences in the CDR3 regions of the BCR heavy and light chains in the Ctrl group of intrathyroidal mononuclear cells are shown as sequence logo plots (Supplementary Figure S7D). Amino acids such as alanine (A), arginine (R), tyrosine (Y), and aspartic acid (D) were prominent in the heavy chain, whereas glutamine (Q), serine (S), and valine (V) were noticeable in the light chain. The amino acid sequences varied between the Ctrl group and the TSHR, Tg, and TPO groups. Similarly, when comparing B cells from peripheral blood and the thyroid, although the CDR3 regions of both the BCR heavy and light chains showed similar patterns, several differences in amino acid sequences were observed.

## 4. Discussion

The following findings were elucidated in this study: 1. After stimulating PBMCs from patients with GD using overlapping peptides of thyroid antigens, the characteristic expression of genes related to B-cell maturation and antibody production, such as *IL6* [18] and *CXCR5* [19], was observed in the naive B-cell cluster. In the memory B-cell cluster, immunoglobulin genes such as *IGHM*, *IGHG2*, *IGHG1*, and *IGHG4*, as well as *CD74*, which binds to MHC class II molecules and regulates antigen presentation [20], were noticeably expressed. 2. The TSHR, Tg, and TPO groups of naive and memory B cells of PBMCs showed the increased expression of MHC class II molecules, such as *HLA-DMA* and *HLA-DRB1* [21,22], compared to the Ctrl group. There was also an upregulation of the expressions of *IGHG*, *IGHM*, *CD74*, and *CD79A*, which form part of the BCR complex and are related to signal transduction [23], and *MS4A1*, which encodes CD20, involved in B-cell maturation and proliferation. 3. In intrathyroidal mononuclear cells, the naive B-cell cluster showed the characteristic expression of *TNFRSF13C*, which is related to B-cell maturation [24], and CD40, which influences B-cell proliferation and differentiation and is associated with GD [6,25]. In the memory B-cell cluster, the expression of *CD79A* and *MS4A1* was characteristic. Furthermore, compared to that in the Ctrl group of PBMC B cells, there was a significant expression of genes related to the suppression of signal transduction, such as *DUSP1*, *DUSP2* [26], and *RGS1* [27], as well as the early activation antigen *CD69* [28], the transcription factor *FOSB* [29], and *IGHG* genes in B cells in the thyroid. 4. In the proteomic analysis, significant differences were observed in the expression of IL-10RB, the receptor of IL-10 that has immunosuppressive effects [30], in both the PBMC and thyroid groups. In the thyroid group, significant differences were observed in IL-7, which is related to B-cell development [31]; IL-18, which is associated with IFN-γ production [32]; and TRAIL, which is involved in apoptosis [33], with particularly notable increases in the TSHR group. 5. In the Ctrl group of naive and memory B cells, *IGHV3-23*, *IGHV4-34*, and *IGHJ4* were frequently observed. The stimulation of PBMCs with TSHR resulted in increased *IGHV4-34/IGHJ4* expression in both naive and memory B-cell clusters. To the best of our knowledge, this is the first report to isolate peripheral and intrathyroidal blood cells from patients with GD, stimulate them with overlapping peptides of thyroid antigens, and analyze them using scRNA-seq, focusing on B cells.

A combination of environmental and genetic factors is associated with GD onset. *CD40* and *PTPN22*, which are involved in signal transduction and influence B-cell proliferation and differentiation, are associated with GD [6,25]. These polymorphisms lead to cytokine secretion, such as IL1-β, IL6, IL12, IL16, IL17, and TNF-α from activated B and T cells, exacerbating thyroid inflammation [6]. Previous scRNA-seq studies evaluating T cells in GD have demonstrated activation of the T-cell receptor signaling pathway, Th1 and Th2 cell differentiation, and chemokine signaling pathways [34]. However, no studies have evaluated gene expression and BCR in GD by stimulating PBMCs and intrathyroidal blood monocytes with thyroid antigens.

Several HLAs have been reported to be risk factors. The dysfunction of HLA-DMA can cause immunodeficiency and autoimmune diseases [35], and HLA-DRB1 has been reported to be associated with the risk of GD and rheumatoid arthritis [22,36,37]. In this study, we considered that HLA-DMA and HLA-DRB1 may also be related to the exacerbation of inflammation in GD. However, the HLA region contributes to only approximately 5% of susceptibility to GD [38,39]. Other immune-related genes, such as *CTLA4*, *CD25*, and *FOXP3*, in addition to *CD40* and *PTPN22*, contribute to susceptibility to GD [36,37,40,41]. Although the risk associated with each of these genes has only a small impact, their interactions and relationships with environmental factors are considered important [1]. In our study, we found that genes related to the immune system and signal transduction, such as *CD40*, *CD74*, *CD79A*, *TNFRSF13C*, *TCF4*, *CXCR5*, and *MS4A1*, were associated with B-cell reactions in GD.

In addition to peripheral blood B cells, intrathyroidal B cells are considered the main source of TRAb because they spontaneously secrete these antibodies despite a decreased proliferative response [42]. In this study, we observed increased expression of immunoglobulin genes in intrathyroidal B cells compared to that in PBMCs, suggesting that the thyroid tissue may be a central site of autoimmune responses against TSHR and may exhibit a stronger immune response. Conversely, an increase in the expression of genes related to the suppression of signal transduction was observed in intrathyroidal B cells. Protein evaluation revealed an increase in IL10RB in both the PBMC and thyroid groups. B cells produce pro-inflammatory cytokines like IL6 but also anti-inflammatory cytokines such as IL10 [43]. Increased IL-10 expression has been reported in autoimmune thyroid diseases [44], suggesting that anti-inflammatory responses may occur simultaneously via IL10RB, DUSP1, DUSP2, and RGS1 in GD.

IGHV3-23, which was frequently observed in this study, is associated with chronic lymphocytic leukemia, has a high mutation load, and interacts with superantigens [45]. IGHV4-34 possesses the characteristic of binding to self-antigens and is commonly seen in the repertoire of naive B cells but is usually eliminated from memory B cells [46]. Therefore, IGHV4-34 is associated with the onset of autoimmune diseases, and a previous analysis of the BCR repertoire in patients with systemic lupus erythematosus has reported increased IGHV4-34 during the acute phases [47]. Thus, IGHV3-23 and IGHV4-34 may be associated with the pathology of GD.

This study had several limitations. First, the patient backgrounds were heterogeneous. In particular, the variability in TRAb and TPOAb levels among GD patients may reflect differing immune profiles, including a mixture of stimulating and blocking antibodies. This immunological heterogeneity should be carefully considered when interpreting the transcriptomic and BCR findings. Second, there was variability in the sample sizes between groups. Third, we could not compare patients with normal thyroid function. Fourth, all PBMCs were included in the proteomic analysis, and we could not only examine B cells. Fifth, the evaluation was based on a gene panel, and a comprehensive investigation was not performed. Furthermore, B cell apoptosis following in vitro stimulation may lead to the selective survival and clonal expansion of specific B cell subsets, potentially introducing bias into the BCR repertoire captured in our study. Finally, subgroup analyses stratified by TRAb or TPOAb levels were not conducted in this study due to the limited sample size.

## 5. Conclusions

In summary, B-cell responses to TSHR, Tg, and TPO differed, and changes in B-cell reactivity were observed between PBMCs and the thyroid. This suggests that in GD inflammation, each thyroid autoantigen exerts different effects on B cells in the peripheral blood or thyroid and is complexly related to the pathology of GD. Additionally, genes such as *IGHV3-23* and *IGHV4-34*, which have been reported to be associated with autoimmune diseases, may also be associated with autoantibody production in GD. Further studies based on these findings are expected to facilitate the development of novel therapeutic strategies.

## Figures and Tables

**Table 1:** Subjects’ profiles.

	Age	Sex	Duration from Onset to Surgery	FT3 (pg/mL)	FT4 (ng/dL)	TSH (μIU/mL)	TRAb (IU/L)	TgAb (IU/mL)	TPOAb (IU/mL)	Preoperative Therapies
Sample1	17	female	6 months	22.2	6.39	<0.005	98.9	18	58	MMI, PTU, KI
Sample2	54	female	32 years	12.1	2.08	<0.005	>50	124	1700	MMI, KI
Sample3	58	female	8 months	27.6	>7.77	<0.005	39	172	4790	MMI, KI
Sample4	48	male	22 years	25.8	2.58	<0.005	>50	1400	7770	MMI, KI, RI
Sample5	21	female	4 months	19.7	6.57	<0.005	3.5	56.2	83.8	MMI, KI
Sample6	18	female	2 years	32.5	>7.77	<0.005	>50	26.4	67.1	MMI, KI
Sample7	45	male	7 years	4.65	2.02	<0.005	3.6	17.4	66.6	MMI, KI
Sample8	26	female	5 years	5.61	2.02	<0.005	3.4	227	2.9	MMI

The blood test data represent the initial consultation at our hospital and may not necessarily reflect the time of disease onset. Abbreviations: FT3, free triiodothyronine; FT4, free thyroxine; TSH: thyroid stimulating hormone; TRAb, thyroid stimulating hormone receptor antibody; TgAb, thyroglobulin antibody; TPOAb, thyroid peroxidase antibody; MMI: methimazole; KI: potassium iodide; PTU: propylthiouracil; RI: radioiodine therapy.

**Table 2:** Top 5 combinations of BCR heavy chain V and J genes and light chain V and J genes for each control group of total B cells, naive B cells, and memory B cells of PBMC.

Total B Cells							
BCR_Heavy_V	BCR_Heavy_J	n	Percentage	BCR_Light_V	BCR_Light_J	n	Percentage
IGHV3-23*01	IGHJ4*02	93	5.973025	IGLV1-44*01	IGLJ3*02	46	2.757794
IGHV3-30*04	IGHJ4*02	38	2.440591	IGKV3-20*01	IGKJ1*01	41	2.458034
IGHV3-48*03	IGHJ4*02	37	2.376365	IGKV1-39*01	IGKJ2*01	40	2.398082
IGHV4-34*01	IGHJ4*02	35	2.247913	IGKV1-5*03	IGKJ1*01	38	2.278177
IGHV4-59*01	IGHJ4*02	32	2.055234	IGKV3-11*01	IGKJ4*01	38	2.278177
Naive B cells							
IGHV3-30*04	IGHJ4*02	8	6.956522	IGKV2-28*01	IGKJ4*01	4	3.361345
IGHV3-23*01	IGHJ4*02	5	4.347826	IGLV1-44*01	IGLJ3*02	4	3.361345
IGHV4-59*01	IGHJ4*02	5	4.347826	IGKV1-5*04	IGKJ1*01	3	2.521008
IGHV3-48*03	IGHJ4*02	4	3.478261	IGKV3-15*01	IGKJ1*01	3	2.521008
IGHV1-3*01	IGHJ3*02	3	2.608696	IGKV3-15*01	IGKJ4*01	3	2.521008
IGHV4-34*01	IGHJ3*02	3	2.608696	IGKV3-20*01	IGKJ1*01	3	2.521008
Memory B cells							
IGHV3-23*01	IGHJ4*02	88	6.102635	IGLV1-44*01	IGLJ3*02	42	2.711427
IGHV4-34*01	IGHJ4*02	34	2.357836	IGKV1-39*01	IGKJ2*01	38	2.453196
IGHV3-48*03	IGHJ4*02	33	2.288488	IGKV3-20*01	IGKJ1*01	38	2.453196
IGHV3-30*04	IGHJ4*02	30	2.080444	IGKV1-5*03	IGKJ1*01	37	2.388638
IGHV3-21*01	IGHJ4*02	29	2.011096	IGKV3-11*01	IGKJ4*01	37	2.388638

Total B cells are the combined of naive and memory B cells. Abbreviations: PBMC: peripheral blood mononuclear cell; BCR: B-cell receptor; n: number.

**Table 3:** (A). Top 5 combinations of BCR heavy chain V and J genes and light chain V and J genes in total B cells in response to overlapping peptides of TSHR, Tg, and TPO antigens and a comparison with the control group of PBMC. (B). Top 5 combinations of BCR heavy chain V and J genes and light chain V and J genes in naive B cells in response to overlapping peptides of TSHR, Tg, and TPO antigens and a comparison with the control group of PBMC. (C). Top 5 combinations of BCR heavy chain V and J genes and light chain V and J genes in memory B cells in response to overlapping peptides of TSHR, Tg, and TPO antigens and a comparison with the control group of PBMC.

(A)													
Total B Cells TSHR Vs. Ctrl													
BCR_Heavy_V	BCR_Heavy_J	n_TSHR	Percentage	n_Ctrl	Percentage	*p*_Value	BCR_Light_V	BCR_Light_J	n_TSHR	Percentage	n_Ctrl	Percentage	*p*_Value
IGHV4-34*01	IGHJ4*02	11	6.9182	35	2.2479	0.0023	IGKV1-39*01	IGKJ2*01	6	3.5294	40	2.3981	0.3109
IGHV3-23*01	IGHJ4*02	7	4.4025	93	5.9730	0.5923	IGKV3-15*01	IGKJ2*01	6	3.5294	20	1.1990	0.0276
IGHV3-30*04	IGHJ4*02	7	4.4025	38	2.4406	0.1839	IGLV1-44*01	IGLJ3*02	6	3.5294	46	2.7578	0.4736
IGHV3-7*01	IGHJ4*02	5	3.1447	21	1.3487	0.0855	IGLV2-14*03	IGLJ3*02	6	3.5294	15	0.8993	0.0096
IGHV4-39*01	IGHJ4*02	5	3.1447	28	1.7983	0.2237	IGKV1-39*01	IGKJ1*01	5	2.9412	25	1.4988	0.1904
							IGKV2-28*01	IGKJ2*01	5	2.9412	21	1.2590	0.0851
Total B cells Tg vs. Ctrl													
BCR_Heavy_V	BCR_Heavy_J	n_Tg	percentage	n_Ctrl	percentage	*p*_value	BCR_Light_V	BCR_Light_J	n_Tg	percentage	n_Ctrl	percentage	*p*_value
IGHV3-23*01	IGHJ4*02	18	7.2289	93	5.9730	0.4764	IGKV3-20*01	IGKJ1*01	16	5.6338	41	2.4580	0.0066
IGHV4-34*01	IGHJ4*02	10	4.0161	35	2.2479	0.1207	IGKV1-39*01	IGKJ2*01	13	4.5775	40	2.3981	0.0467
IGHV3-30*04	IGHJ3*02	7	2.8112	5	0.3211	0.0004	IGKV1-39*01	IGKJ1*01	9	3.1690	25	1.4988	0.0796
IGHV3-30*04	IGHJ4*02	7	2.8112	38	2.4406	0.6638	IGKV1-5*03	IGKJ1*01	8	2.8169	38	2.2782	0.5288
IGHV4-39*01	IGHJ4*02	7	2.8112	28	1.7983	0.3168	IGLV2-14*01	IGLJ3*02	8	2.8169	36	2.1583	0.5142
Total B cells TPO vs. Ctrl													
BCR_Heavy_V	BCR_Heavy_J	n_TPO	percentage	n_Ctrl	percentage	*p*_value	BCR_Light_V	BCR_Light_J	n_TPO	percentage	n_Ctrl	percentage	*p*_value
IGHV3-23*01	IGHJ4*02	30	5.0590	93	5.9730	0.4675	IGKV1-39*01	IGKJ2*01	28	4.628099	40	2.398082	0.0079
IGHV4-34*01	IGHJ4*02	18	3.0354	35	2.2479	0.2806	IGKV3-20*01	IGKJ1*01	19	3.140496	41	2.458034	0.3758
IGHV3-30*04	IGHJ4*02	16	2.6981	38	2.4406	0.7582	IGLV1-44*01	IGLJ3*02	18	2.975207	46	2.757794	0.7751
IGHV3-48*03	IGHJ4*02	14	2.3609	37	2.3764	1.0000	IGKV1-39*01	IGKJ1*01	18	2.975207	25	1.498801	0.0347
IGHV4-39*01	IGHJ4*02	13	2.1922	28	1.7983	0.5967	IGKV3-20*01	IGKJ2*01	16	2.644628	33	1.978417	0.3298
							IGKV1-39*01	IGKJ4*01	16	2.644628	17	1.019185	0.0084
(B)													
Naive Bcell TSHR Vs. Ctrl													
BCR_Heavy_V	BCR_Heavy_J	n_TSHR	Percentage	n_Ctrl	Percentage	*p*_Value	BCR_Light_V	BCR_Light_J	n_TSHR	Percentage	n_Ctrl	Percentage	*p*_Value
IGHV4-34*01	IGHJ4*02	7	6.3636	1	0.8696	0.0324	IGLV2-14*03	IGLJ3*02	6	5.1724	1	0.8403	0.0635
IGHV3-30*04	IGHJ4*02	6	5.4545	8	6.9565	0.7845	IGKV1-39*01	IGKJ1*01	5	4.3103	2	1.6807	0.2765
IGHV3-23*01	IGHJ4*02	5	4.5455	5	4.3478	1.0000	IGKV1-39*01	IGKJ2*01	4	3.4483	2	1.6807	0.4421
IGHV4-39*01	IGHJ4*02	4	3.6364	2	1.7391	0.4378	IGKV2-28*01	IGKJ2*01	4	3.4483	2	1.6807	0.4421
IGHV1-18*01	IGHJ4*02	3	2.7273	0	0.0000	0.1152	IGLV3-19*01	IGLJ2*01	4	3.4483	1	0.8403	0.2089
IGHV1-69*02	IGHJ4*02	3	2.7273	1	0.8696	0.3608							
Naive Bcell Tg Vs. Ctrl													
BCR_Heavy_V	BCR_Heavy_J	n_Tg	percentage	n_Ctrl	percentage	*p*_value	BCR_Light_V	BCR_Light_J	n_Tg	percentage	n_Ctrl	percentage	*p*_value
IGHV3-23*01	IGHJ4*02	12	7.5949	5	4.3478	0.3194	IGKV3-20*01	IGKJ1*01	11	5.8511	3	2.5210	0.2618
IGHV4-34*01	IGHJ4*02	7	4.4304	1	0.8696	0.1442	IGLV1-51*01	IGLJ2*01	7	3.7234	2	1.6807	0.4904
IGHV3-30*04	IGHJ4*02	6	3.7975	8	6.9565	0.2748	IGKV1-39*01	IGKJ1*01	6	3.1915	2	1.6807	0.4912
IGHV3-30*04	IGHJ3*02	5	3.1646	1	0.8696	0.4062	IGKV1-39*01	IGKJ2*01	6	3.1915	2	1.6807	0.4912
IGHV4-39*01	IGHJ4*02	5	3.1646	2	1.7391	0.7026	IGKV1-5*03	IGKJ1*01	6	3.1915	1	0.8403	0.2547
Naive Bcell TPO Vs. Ctrl													
BCR_Heavy_V	BCR_Heavy_J	n_TPO	percentage	n_Ctrl	percentage	*p*_value	BCR_Light_V	BCR_Light_J	n_TPO	percentage	n_Ctrl	percentage	*p*_value
IGHV3-23*01	IGHJ4*02	20	5.1282	5	4.3478	1.0000	IGKV1-39*01	IGKJ2*01	18	4.5802	2	1.6807	0.1856
IGHV4-34*01	IGHJ4*02	14	3.5897	1	0.8696	0.2093	IGKV1-39*01	IGKJ1*01	15	3.8168	2	1.6807	0.3829
IGHV3-30*04	IGHJ4*02	12	3.0769	8	6.9565	0.0968	IGKV3-20*01	IGKJ1*01	14	3.5623	3	2.5210	0.7732
IGHV3-48*03	IGHJ4*02	11	2.8205	4	3.4783	0.7551	IGLV1-44*01	IGLJ3*02	12	3.0534	4	3.3613	0.7718
IGHV3-30*18	IGHJ4*02	10	2.5641	0	0.0000	0.1264	IGKV1-39*01	IGKJ4*01	10	2.5445	1	0.8403	0.4707
(C)													
Memory Bcell TSHR Vs. Ctrl													
BCR_Heavy_V	BCR_Heavy_J	n_TSHR	Percentage	n_Ctrl	Percentage	*p*_Value	BCR_Light_V	BCR_Light_J	n_TSHR	Percentage	n_Ctrl	Percentage	*p*_Value
IGHV4-34*01	IGHJ4*02	4	8.1633	34	2.3578	0.0334	IGKV3-15*01	IGKJ2*01	3	5.5556	18	1.1620	0.0313
IGHV4-39*07	IGHJ4*02	3	6.1224	14	0.9709	0.0164	IGLV1-44*01	IGLJ3*02	3	5.5556	42	2.7114	0.1911
IGHV3-23*01	IGHJ4*02	2	4.0816	88	6.1026	0.7649	IGLV2-14*01	IGLJ1*01	3	5.5556	21	1.3557	0.0444
IGHV3-49*04	IGHJ4*02	2	4.0816	11	0.7628	0.0655	IGLV6-57*04	IGLJ3*02	3	5.5556	0	0.0000	0.0000
IGHV3-7*01	IGHJ4*02	2	4.0816	20	1.3870	0.1613	IGKV1-39*01	IGKJ2*01	2	3.7037	38	2.4532	0.3934
IGHV3-74*01	IGHJ4*02	2	4.0816	15	1.0402	0.1052	IGKV3-11*01	IGKJ1*01	2	3.7037	8	0.5165	0.0422
Memory Bcell Tg vs. Ctrl													
BCR_Heavy_V	BCR_Heavy_J	n_Tg	percentage	n_Ctrl	percentage	*p*_value	BCR_Light_V	BCR_Light_J	n_Tg	percentage	n_Ctrl	percentage	*p*_value
IGHV3-23*01	IGHJ4*02	6	6.5934	88	6.1026	0.8209	IGKV1-39*01	IGKJ2*01	7	7.2917	38	2.4532	0.0135
IGHV1-18*01	IGHJ4*02	4	4.3956	14	0.9709	0.0187	IGKV3-20*01	IGKJ1*01	5	5.2083	38	2.4532	0.1012
IGHV3-33*01	IGHJ4*02	3	3.2967	12	0.8322	0.0548	IGKV3-15*01	IGKJ1*01	4	4.1667	31	2.0013	0.1432
IGHV4-34*01	IGHJ4*02	3	3.2967	34	2.3578	0.4794	IGLV2-14*01	IGLJ3*02	4	4.1667	34	2.1950	0.2764
IGHV3-23*03	IGHJ6*02	2	2.1978	7	0.4854	0.0956	IGKV1-39*01	IGKJ1*01	3	3.1250	23	1.4848	0.1906
IGHV3-30*04	IGHJ3*02	2	2.1978	4	0.2774	0.0447	IGKV1-8*02	IGKJ5*01	3	3.1250	2	0.1291	0.0018
Memory Bcell TPO vs. Ctrl													
BCR_Heavy_V	BCR_Heavy_J	n_TPO	percentage	n_Ctrl	percentage	*p*_value	BCR_Light_V	BCR_Light_J	n_TPO	percentage	n_Ctrl	percentage	*p*_value
IGHV3-23*01	IGHJ4*02	10	4.9261	88	6.1026	0.6345	IGKV1-39*01	IGKJ2*01	10	4.7170	38	2.4532	0.0696
IGHV4-39*01	IGHJ4*02	5	2.4631	26	1.8031	0.5770	IGKV4-1*01	IGKJ4*01	8	3.7736	24	1.5494	0.0470
IGHV3-30*04	IGHJ4*02	4	1.9704	30	2.0804	1.0000	IGKV3-20*01	IGKJ2*01	7	3.3019	32	2.0658	0.3136
IGHV4-34*01	IGHJ4*02	4	1.9704	34	2.3578	1.0000	IGLV2-14*01	IGLJ3*02	7	3.3019	34	2.1950	0.3274
IGHV5-51*01	IGHJ4*02	4	1.9704	23	1.5950	0.5667	IGKV1-39*01	IGKJ4*01	6	2.8302	16	1.0329	0.0402
							IGKV3-15*01	IGKJ2*01	6	2.8302	18	1.1620	0.0592

Abbreviations: PBMC: peripheral blood mononuclear cell; BCR: B-cell receptor; TSHR: thyroid-stimulating hormone receptor; Tg, thyroglobulin; TPO, thyroid peroxidase. Total B cells are the combined of naive and memory B cells. Data were analyzed by using Fisher’s exact test.

## Data Availability

The high-throughput sequencing data generated in this study have been deposited in the GEO database under accession number GSE285196 (https://www.ncbi.nlm.nih.gov/geo/query/acc.cgi?acc=GSE285196). These data were made publicly available on 23 December 2024.

## Supplementary Materials

The following supporting information can be downloaded at: https://www.mdpi.com/article/10.3390/cells14141102/s1, Figure S1: Cluster identification and gene expression in B-cell clusters of PBMCs. (A) Cluster identification of PBMCs by antigen. (B) Cluster identification of lymphoid cells in PBMCs by antigen. (C) Dot plots for the expression of the top 5 marker genes in each cluster of lymphoid cells in PBMCs. (D) Violin plot of genes related to cytokine in naive B-cell clusters in PBMCs by antigen. (E) Violin plot of genes related to cytokine in memory B-cell clusters in PBMCs by antigen. (F) Violin plot of TNFRSF17 in memory B-cell clusters in PBMCs by antigen. PBMC: peripheral blood mononuclear cells; TSHR: thyroid-stimulating hormone receptor; Tg: thyroglobulin; TPO: thyroid peroxidase.; Figure S2: Cluster identification of intrathyroidal blood mononuclear cells. (A) Cluster identification of intrathyroidal blood mononuclear cells by antigen. (B) Cluster identification of intrathyroidal lymphoid cells by antigen. (C) Dot plots for the expression of the top 5 marker genes in each cluster of intrathyroidal lymphoid cells. TSHR: thyroid-stimulating hormone receptor; Tg: thyroglobulin; TPO: thyroid Peroxidase. Figure S3: Concentrations of proteins associated with inflammation are produced by blood mono-nuclear cells upon stimulation with each antigen. (A) Genes with significant differences in protein concentrations produced by PBMCs upon stimulation with each antigen. (B) Genes with significant differences in protein concentrations produced by intrathyroidal blood mononuclear cells upon stimulation with each antigen. Data were presented as medians and interquartile ranges (IQR). Data were analyzed using one-way ANOVA with Tukey’s honest significant difference test. *: *p* < 0.05. PBMC: peripheral blood mononuclear cell. Figure S4: Bubble plot showing BCR repertoires of total B cells in PBMCs. (A) Bubble plots showing BCR repertoires of total B cells in PBMCs for the control group. (B) Bubble plots showing BCR repertoires of total B cells in PBMCs for the TSHR group. (C) Bubble plots showing BCR repertoires of total B cells in PBMCs for the Tg group. (D) Bubble plots showing BCR repertoires of total B cells in PBMCs for the TPO group. PBMC: peripheral blood mononuclear cells; BCR: B-cell receptor; TSHR: thyroidstimulating hormone receptor; Tg: thyroglobulin; TPO: thyroid peroxidase; Figure S5:Bubble plot showing BCR repertoires of naive B cells in PBMCs. (A) Bubble plots showing BCR repertoires of naive B cells in PBMCs for the control group. (B) Bubble plots showing BCR repertoires of naive B cells in PBMCs for the TSHR group. (C) Bubble plots showing BCR repertoires of naive B cells in PBMCs for the Tg group. (D) Bubble plots showing BCR repertoires of naive B cells in PBMCs for the TPO group. PBMC: peripheral blood mononuclear cells; BCR: B-cell receptor; TSHR: thyroidstimulating hormone receptor; Tg: thyroglobulin; TPO: thyroid peroxidase; Figure S6: Bubble plot showing BCR repertoires of memory B cells in PBMCs. (A) Bubble plots showing BCR repertoires of memory B cells in PBMCs for the control group. (B) Bubble plots showing BCR repertoires of memory B cells in PBMCs for the TSHR group. (C) Bubble plots showing BCR repertoires of memory B cells in PBMCs for the Tg group. (D) Bubble plots showing BCR repertoires of memory B cells in PBMCs for the TPO group. PBMC: peripheral blood mononuclear cells; BCR: B-cell receptor; TSHR: thyroidstimulating hormone receptor; Tg: thyroglobulin; TPO: thyroid peroxidase.; Figure S7. Sequence logo plots of CDR3 motifs of B-cell receptor. (A) Sequence logo plots of BCR heavy and light chain CDR3 motifs in total B cells from PBMCs, grouped by antigen. (B) Sequence logo plots of BCR heavy and light chain CDR3 motifs in naive B cells from PBMCs, grouped by antigen. (C) Sequence logo plots of BCR heavy and light chain CDR3 motifs in memory B cells from PBMCs, grouped by antigen. (D) Sequence logo plots of BCR heavy and light chain CDR3 motifs in the control group of B cells from the thyroid. PBMC: peripheral blood mononuclear cell; BCR: B-cell receptor. TSHR: thyroidstimulating hormone receptor; Tg: thyroglobulin; TPO: thyroid peroxidase. Amino acid abbreviations: A, alanine; R, arginine; N, asparagine; D, aspartic acid; C, cysteine; Q, glutamine; E, glutamic acid; G, glycine; H, histidine; I, isoleucine; L, leucine; K, lysine; M, methionine; F, phenylalanine; P, proline; S, serine; T, threonine; W, tryptophan; Y, tyrosine; V, va-line. Table S1: List of overlapping peptide sequences; Table S2A. Gene expression changes in total B cells of PBMC in response to overlapping peptides of TSHR, Tg, and TPO antigens compared to control; Table S2B. Gene expression changes in naive B cells of PBMC in response to overlapping peptides of TSHR, Tg, and TPO antigens compared to control; Table S2C. Gene expression changes in memory B cells of PBMC in response to overlapping peptides of TSHR, Tg, and TPO antigens compared to control.
