# Correction: Kobayashi et al. Pathogenesis of Graves’ Disease Determined Using Single-Cell Sequencing with Thyroid Autoantigen Peptide Stimulation in B Cells. *Cells* 2025, *14*, 1102

**DOI:** 10.3390/cells15090751

**Published:** 2026-04-23

**Authors:** Genki Kobayashi, Takuro Okamura, Yoshitaka Hashimoto, Kimiko Sakai, Madoka Sumi, Dan Imai, Nobuko Kitagawa, Masahide Hamaguchi, Takahiro Tsujikawa, Shigeru Hirano, Michiaki Fukui

**Affiliations:** 1 Department of Endocrinology and Metabolism, Graduate School of Medical Science, Kyoto Prefectural University of Medicine, Kyoto 602-8566, Japan; genkoba@koto.kpu-m.ac.jp (G.K.); d04sm012@koto.kpu-m.ac.jp (T.O.); k-sakai@koto.kpu-m.ac.jp (K.S.); sumi0220@koto.kpu-m.ac.jp (M.S.); dimai@koto.kpu-m.ac.jp (D.I.); nobuko-s@koto.kpu-m.ac.jp (N.K.); michiaki@koto.kpu-m.ac.jp (M.F.); 2 Department of Diabetes and Endocrinology, Matsushita Memorial Hospital, Moriguchi 570-8540, Japan; y-hashi@koto.kpu-m.ac.jp; 3 Department of Otolaryngology–Head and Neck Surgery, Graduate School of Medical Science, Kyoto Prefectural University of Medicine, Kyoto 602-8566, Japan; tu-ji@koto.kpu-m.ac.jp (T.T.); hirano@koto.kpu-m.ac.jp (S.H.)

Takahiro Tsujikawa and Shigeru Hirano were not included as authors in the original publication [1]. The authors declare no conflicts of interest. The corrected Author Contributions statement appears below. The authors state that the scientific conclusions are unaffected. This correction was approved by the Academic Editor. The original publication has also been updated.

**Author Contributions:** Conceptualization: T.O., M.H. and M.F. Methodology: T.O., M.H. and M.F. Validation: G.K., T.O., Y.H., M.H. and M.F. Formal Analysis: G.K., T.O. and M.H. Investigation: G.K., T.O., Y.H., K.S., M.S., D.I., N.K., T.T., S.H., M.H. and M.F. Resources: T.T. and S.H. Data Curation: G.K. and T.O. Writing—Original Draft Preparation: G.K. and T.O. Writing—Review and Editing: T.T., S.H., M.H. and M.F. Visualization: G.K. and T.O. Supervision: T.O., M.H. and M.F. Project Administration: T.O. and M.H. Funding Acquisition: T.O., Y.H. and M.H. All authors have read and agreed to the published version of the manuscript.
