# Supplementary material for: Pathogenesis of Graves’ Disease Determined Using Single-Cell Sequencing with Thyroid Autoantigen Peptide Stimulation in B Cells
Source: Cells. 2025 Jul 17;14(14):1102. doi: 10.3390/cells14141102 (PMC12293664; doi:10.3390/cells14141102)
Supplement: Supplementary file 1 [file cells-14-01102-s001.zip › cells-3648858-supplementary.pdf]

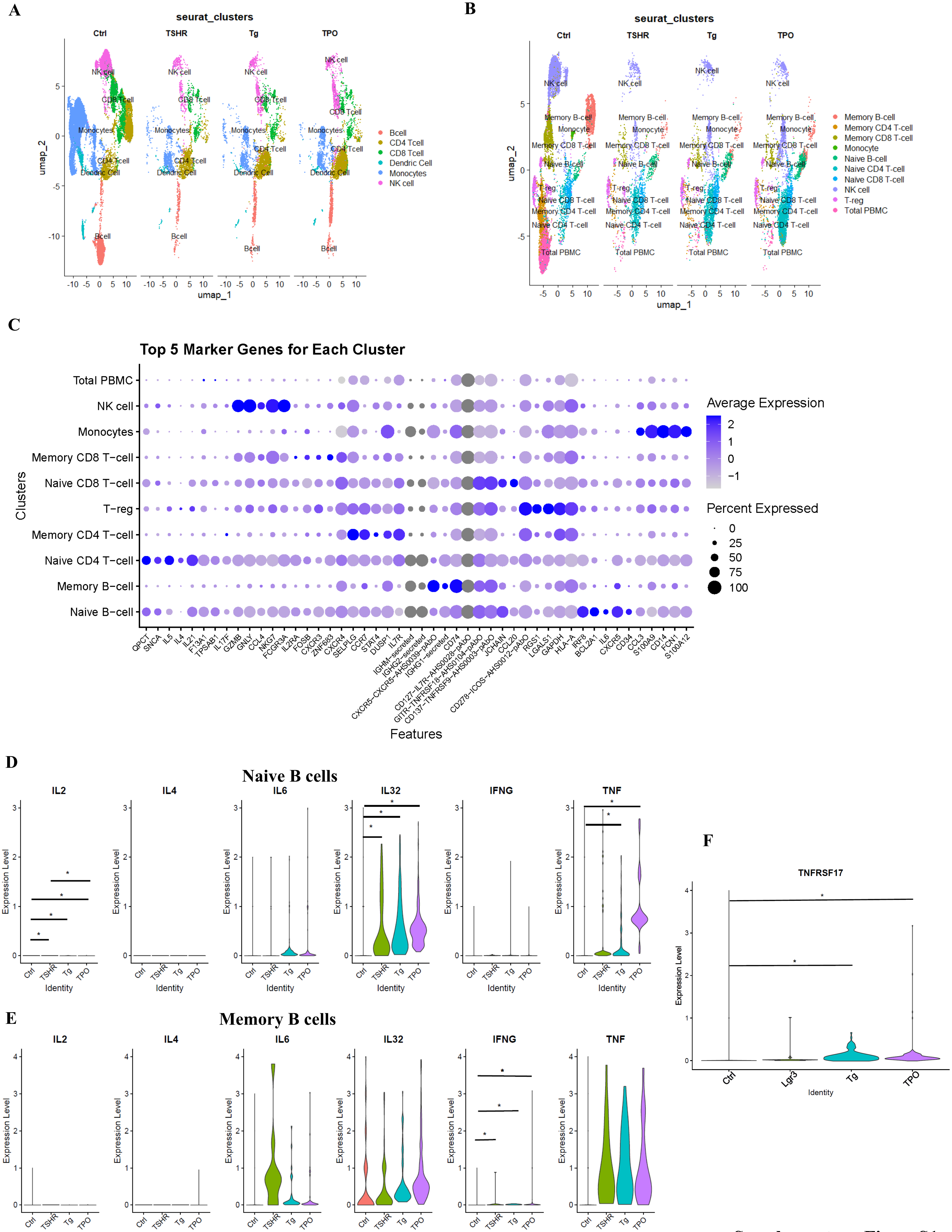

Supplementary FigureS1

Supplementary Figure S1. Cluster identification and gene expression in B-cell clusters of PBMCs.

(A) Cluster identification of PBMCs by antigen. **(B)** Cluster identification of lymphoid cells in PBMCs by antigen. **(C)** Dot plots for the expression of the top 5 marker genes in each cluster of lymphoid cells in PBMCs. **(D)** Violin plot of genes related to cytokine in naive B-cell clusters in PBMCs by antigen. **(E)** Violin plot of genes related to cytokine in memory B-cell clusters in PBMCs by antigen. **(F)** Violin plot of TNFRSF17 in memory B-cell clusters in PBMCs by antigen.

PBMC: peripheral blood mononuclear cells; TSHR: thyroid-stimulating hormone receptor; Tg: thyroglobulin; TPO: thyroid peroxidase.

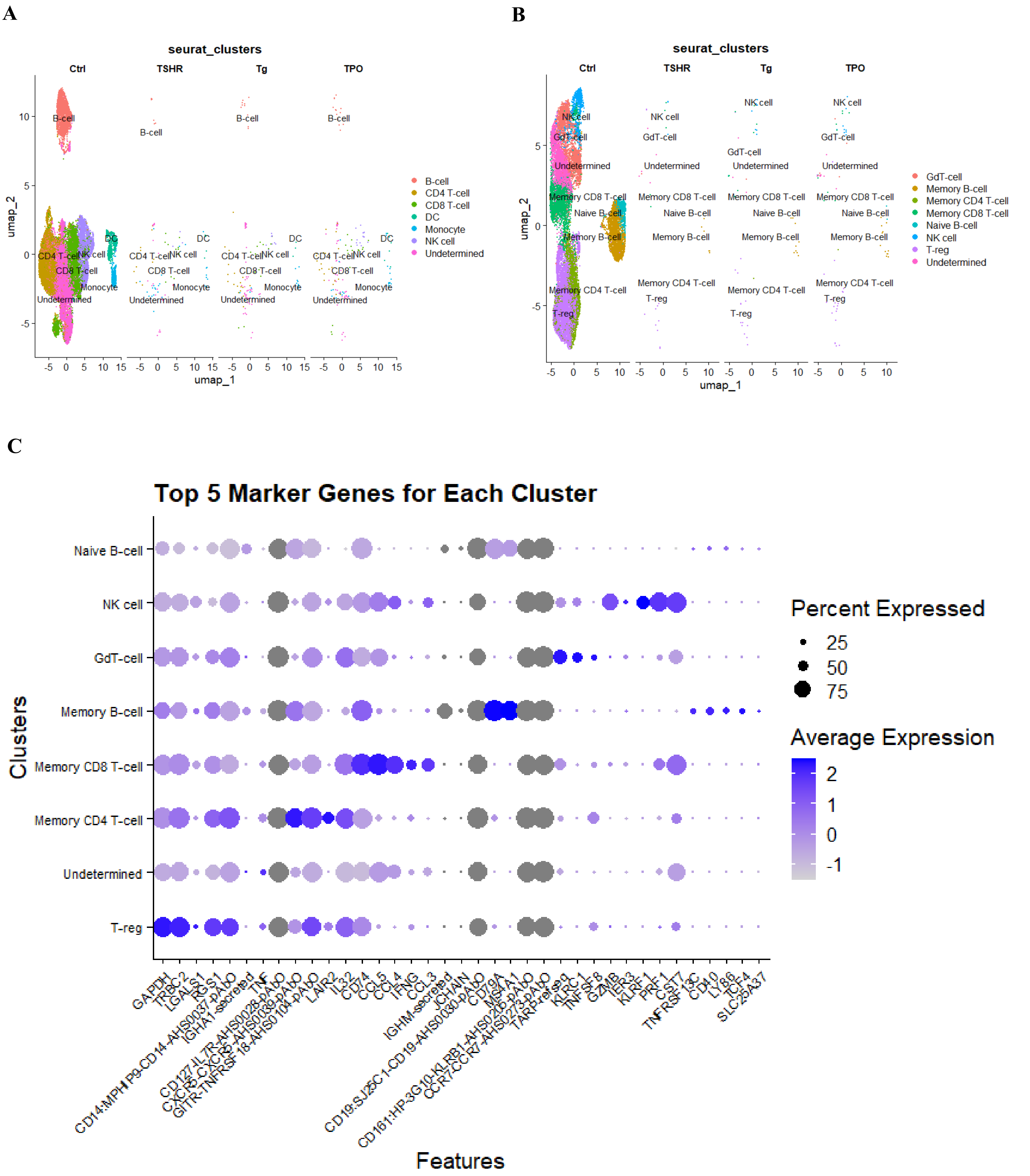

Supplementary FigureS2

Supplementary Figure S2. Cluster identification of intrathyroidal blood mononuclear cells.

(A) Cluster identification of intrathyroidal blood mononuclear cells by antigen. **(B)** Cluster identification of intrathyroidal lymphoid cells by antigen. **(C)** Dot plots for the expression of the top 5 marker genes in each cluster of intrathyroidal lymphoid cells.

TSHR: thyroid-stimulating hormone receptor; Tg: thyroglobulin; TPO: thyroid peroxidase.

A

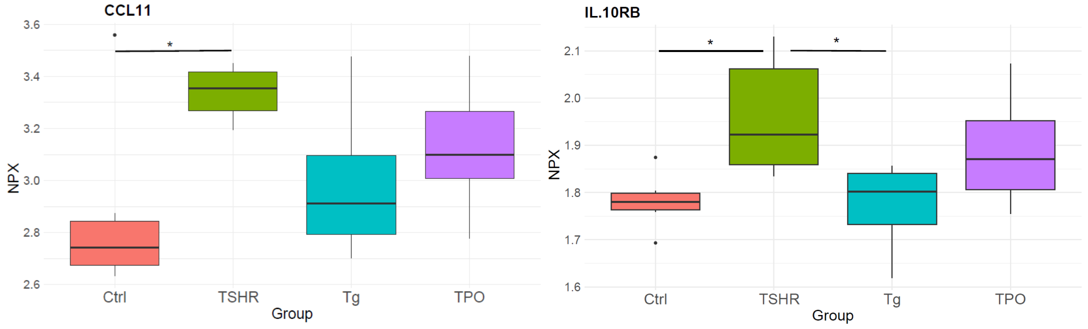

B

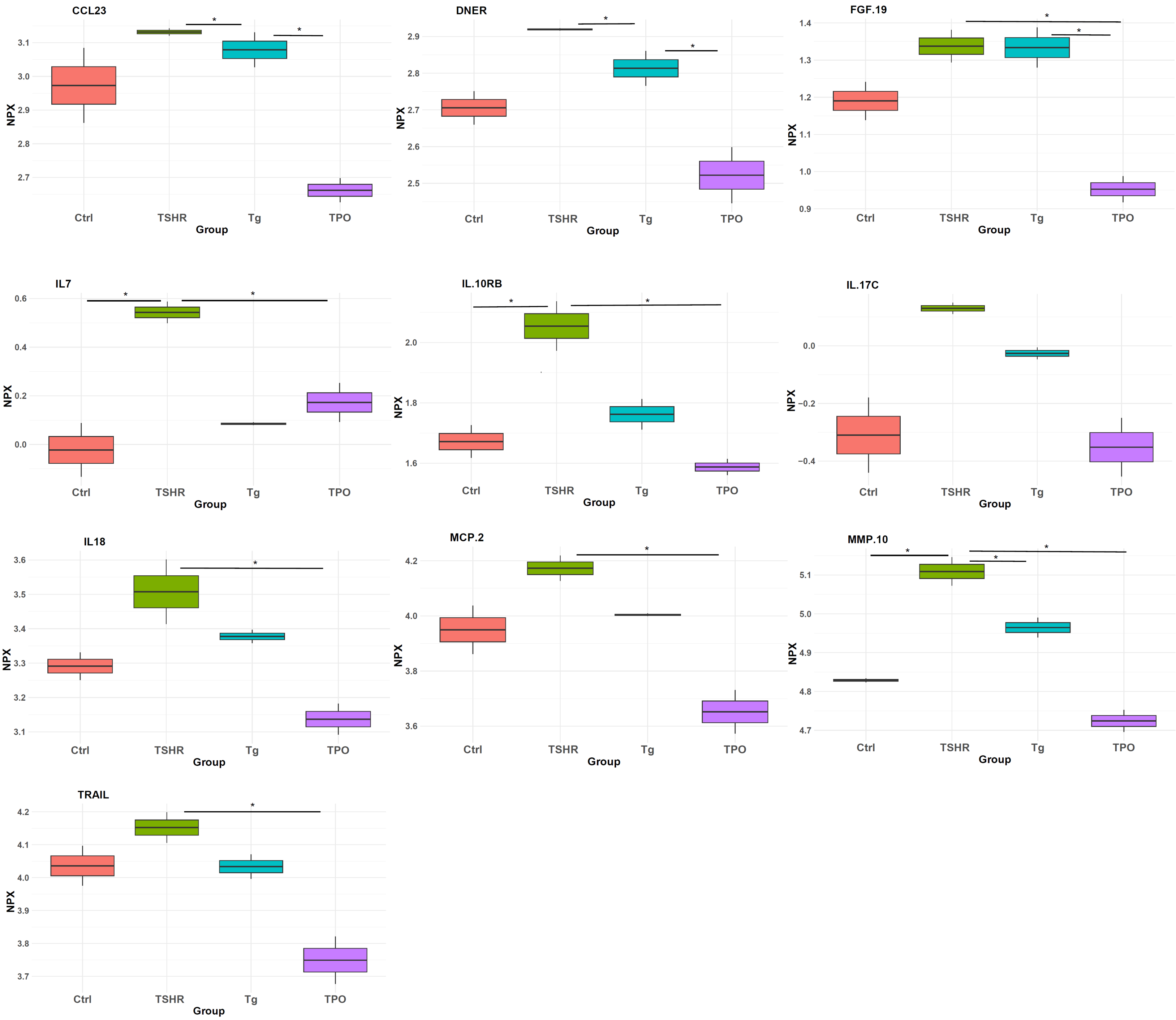

Supplementary FigureS3

Supplementary Figure S3. Concentrations of proteins associated with inflammation are produced by blood mono-nuclear cells upon stimulation with each antigen.

(A) Genes with significant differences in protein concentrations produced by PBMCs upon stimulation with each antigen. **(B)** Genes with significant differences in protein concentrations produced by intrathyroidal blood mononuclear cells upon stimulation with each antigen. Data were presented as medians and interquartile ranges (IQR). Data were analyzed using one-way ANOVA with Tukey's honest significant difference test. *: $p < 0.05$. PBMC: peripheral blood mononuclear cell.

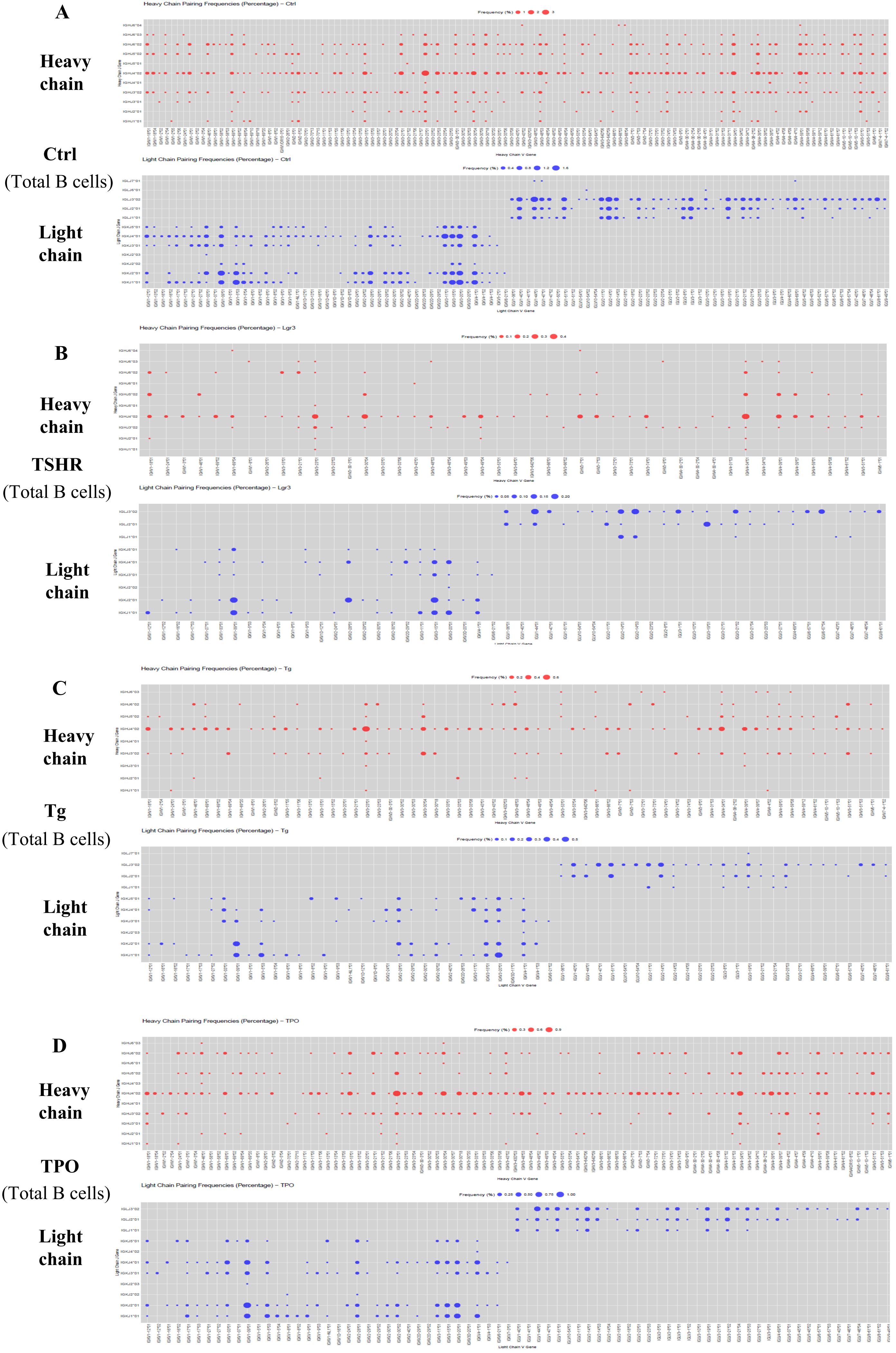

Supplementary Figure S4

Supplementary Figure S4. Bubble plot showing BCR repertoires of total B cells in PBMCs

(A) Bubble plots showing BCR repertoires of total B cells in PBMCs for the control group. **(B)** Bubble plots showing BCR repertoires of total B cells in PBMCs for the TSHR group. **(C)** Bubble plots showing BCR repertoires of total B cells in PBMCs for the Tg group. **(D)** Bubble plots showing BCR repertoires of total B cells in PBMCs for the TPO group.

PBMC: peripheral blood mononuclear cells; BCR: B-cell receptor; TSHR: thyroid-stimulating hormone receptor; Tg: thyroglobulin; TPO: thyroid peroxidase.

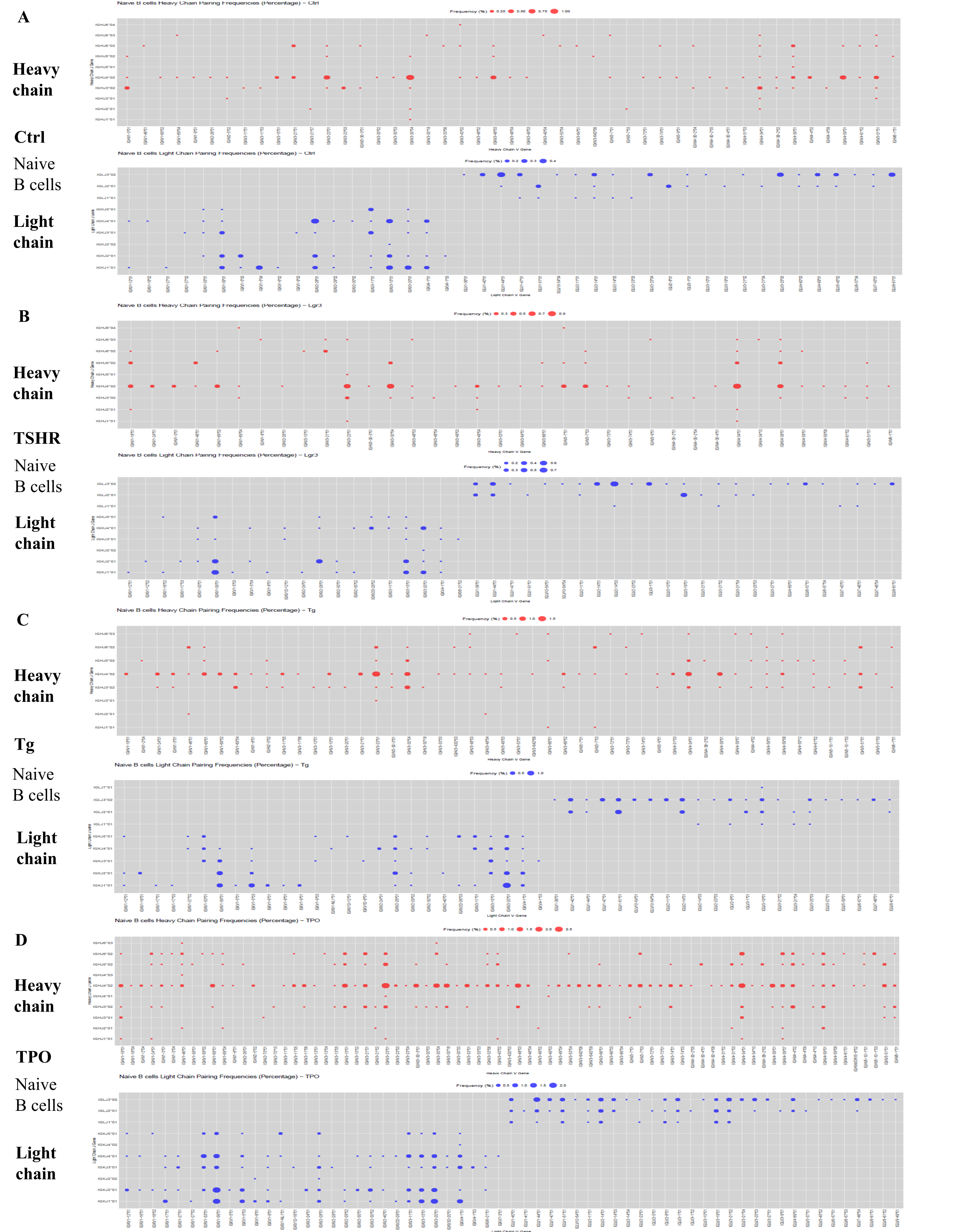

Supplementary Figure S5

Supplementary Figure S5. Bubble plot showing BCR repertoires of naive B cells in PBMCs

(A) Bubble plots showing BCR repertoires of naive B cells in PBMCs for the control group. **(B)** Bubble plots showing BCR repertoires of naive B cells in PBMCs for the TSHR group. **(C)** Bubble plots showing BCR repertoires of naive B cells in PBMCs for the Tg group. **(D)** Bubble plots showing BCR repertoires of naive B cells in PBMCs for the TPO group.

PBMC: peripheral blood mononuclear cells; BCR: B-cell receptor; TSHR: thyroid-stimulating hormone receptor; Tg: thyroglobulin; TPO: thyroid peroxidase.

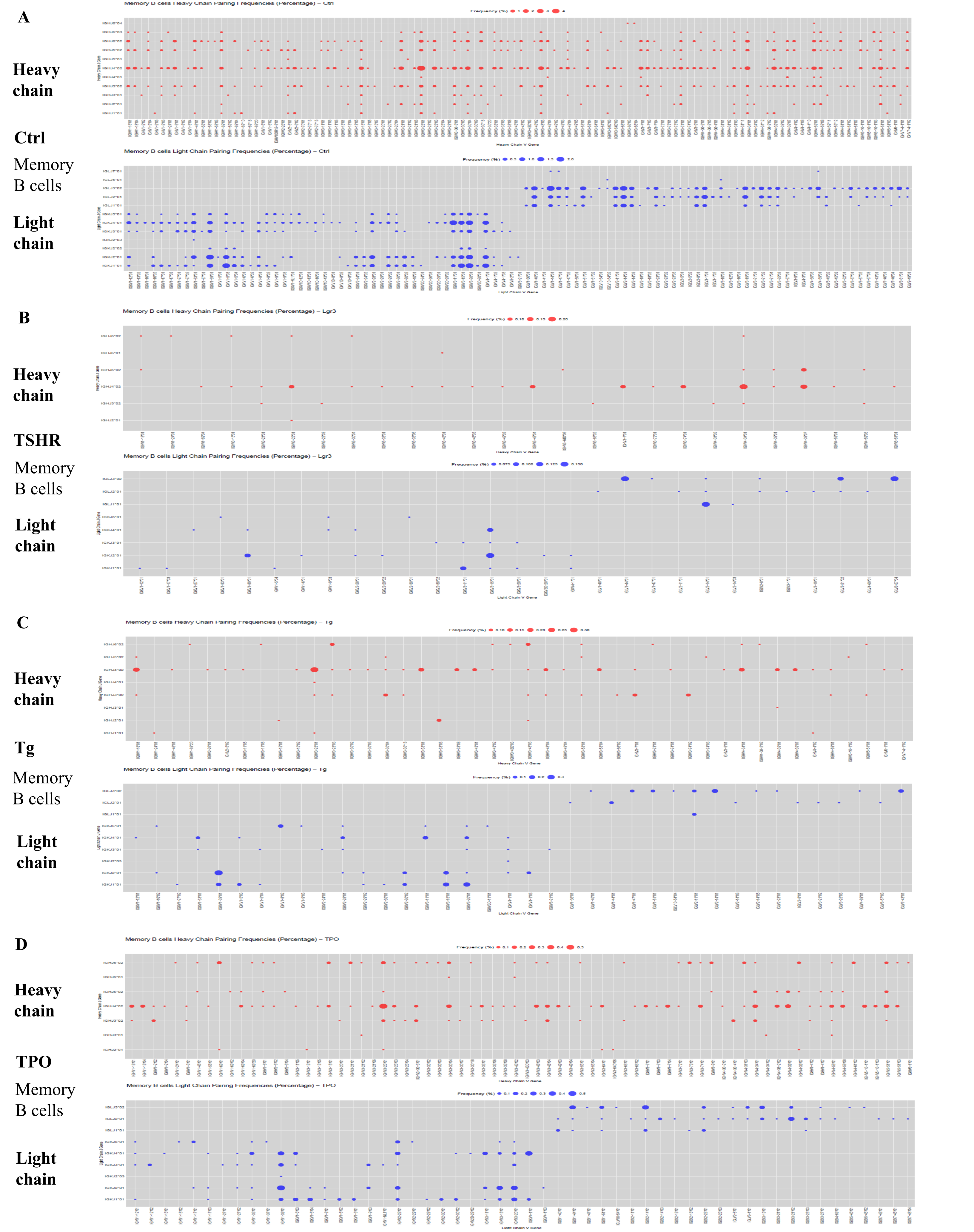

Supplementary Figure S6

Supplementary Figure S6. Bubble plot showing BCR repertoires of memory B cells in PBMCs

(A) Bubble plots showing BCR repertoires of memory B cells in PBMCs for the control group. **(B)** Bubble plots showing BCR repertoires of memory B cells in PBMCs for the TSHR group. **(C)** Bubble plots showing BCR repertoires of memory B cells in PBMCs for the Tg group. **(D)** Bubble plots showing BCR repertoires of memory B cells in PBMCs for the TPO group.

PBMC: peripheral blood mononuclear cells; BCR: B-cell receptor; TSHR: thyroid-stimulating hormone receptor; Tg: thyroglobulin; TPO: thyroid peroxidase.

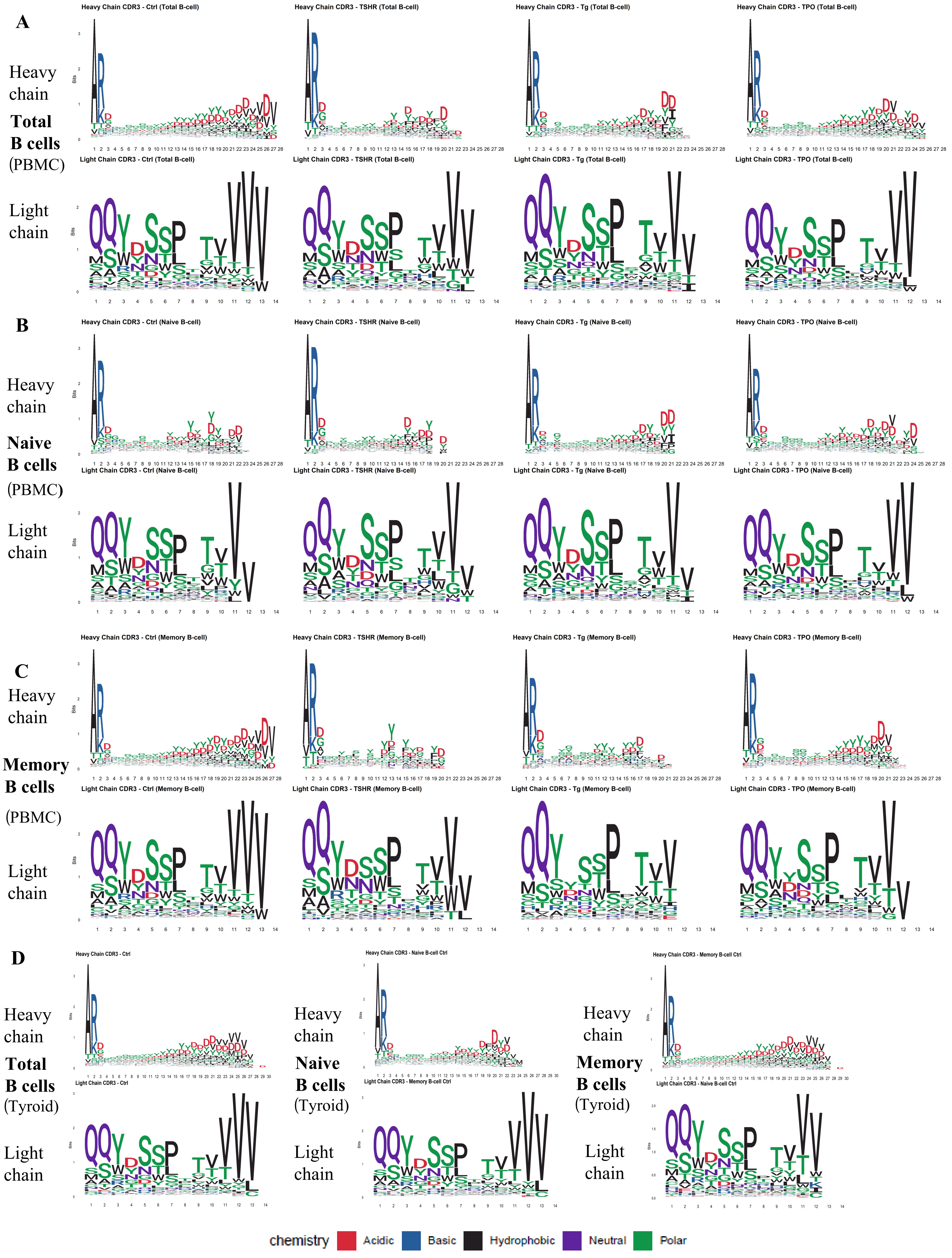

Supplementary FigureS7

Supplementary Figure S7. Sequence logo plots of CDR3 motifs of B-cell receptor.

(A) Sequence logo plots of BCR heavy and light chain CDR3 motifs in total B cells from PBMCs, grouped by antigen. **(B)** Sequence logo plots of BCR heavy and light chain CDR3 motifs in naive B cells from PBMCs, grouped by antigen. **(C)** Sequence logo plots of BCR heavy and light chain CDR3 motifs in memory B cells from PBMCs, grouped by antigen. **(D)** Sequence logo plots of BCR heavy and light chain CDR3 motifs in the control group of B cells from the thyroid.

PBMC: peripheral blood mononuclear cell; BCR: B-cell receptor. TSHR: thyroid-stimulating hormone receptor; Tg: thyroglobulin; TPO: thyroid peroxidase.

Amino acid abbreviations: A, alanine; R, arginine; N, asparagine; D, aspartic acid; C, cysteine; Q, glutamine; E, glutamic acid; G, glycine; H, histidine; I, isoleucine; L, leucine; K, lysine; M, methionine; F, phenylalanine; P, proline; S, serine; T, threonine; W, tryptophan; Y, tyrosine; V, va-line.

Seq1	TSHR				Seq1	Tg				Seq1	TPO			
Nr	Normal Scan	Start	End	Overlap	Nr	Normal Scan	Start	End	Overlap	Nr	Normal Scan	Start	End	Overlap
1	GMGCSSPPCECHQEE	1	15	0	1	MSPFLYLVLLVLGLH	1	15	0	1	MRALAVLSVTLVMAC	1	15	0
2	SPPCECHQEEDFRVT	5	19	11	2	YLVLLVLGLHATIHC	5	19	11	2	VLSVTLVMACTEAFF	5	19	11
3	CHQEEDFRVTCKDIQ	9	23	11	3	VLGLHATIHCASPEG	9	23	11	3	LVMACTEAFFPFISR	9	23	11
4	DFRVTCKDIQRIPSL	13	27	11	4	ATIHCASPEGKVTAC	13	27	11	4	TEAFFPFISRGKELL	13	27	11
5	CKDIQRIPSLPPSTQ	17	31	11	5	ASPEGKVTACHSSQP	17	31	11	5	PFISRGKELLWGKPE	17	31	11
6	RIPSLPPSTQTLKLI	21	35	11	6	KVTACHSSQPNATLY	21	35	11	6	GKELLWGKPEESRVS	21	35	11
7	PPSTQTLKLIETHLR	25	39	11	7	HSSQPNATLYKMSSI	25	39	11	7	WGKPEESRVSSVLEE	25	39	11
8	TLKLIETHLRTIPSH	29	43	11	8	NATLYKMSSINADFA	29	43	11	8	ESRVSSVLEESKRLV	29	43	11
9	ETHLRTIPSHAFSNL	33	47	11	9	KMSSINADFAFNLYR	33	47	11	9	SVLEESKRLVDTAMY	33	47	11
10	TIPSHAFSNLPNISR	37	51	11	10	NADFAFNLYRRFTVE	37	51	11	10	SKRLVDTAMYATMQR	37	51	11
11	AFSNLPNISRIYVSI	41	55	11	11	FNLYRRFTVETPDKN	41	55	11	11	DTAMYATMQRNLKKR	41	55	11
12	PNISRIYVSIDVTLQ	45	59	11	12	RFTVETPDKNIFFSP	45	59	11	12	ATMQRNLKKRGILSP	45	59	11
13	IYVSIDVTLQQLESH	49	63	11	13	TPDKNIFFSPVSISA	49	63	11	13	NLKKRGILSPAQLLS	49	63	11
14	DVTLQQLESHSFYNL	53	67	11	14	IFFSPVSISAALVML	53	67	11	14	GILSPAQLLSFSKLP	53	67	11
15	QLESHSFYNLSKVTH	57	71	11	15	VSISAALVMLSFGAC	57	71	11	15	AQLLSFSKLPEPTSG	57	71	11
16	SFYNLSKVTHIEIRN	61	75	11	16	ALVMLSFGACCSTQT	61	75	11	16	FSKLPEPTSGVIARA	61	75	11
17	SKVTHIEIRNTRNLT	65	79	11	17	SFGACCSTQTEIVET	65	79	11	17	EPTSGVIARAAEIME	65	79	11
18	IEIRNTRNLTYIDPD	69	83	11	18	CSTQTEIVETLGFNL	69	83	11	18	VIARAAEIMETSIQA	69	83	11
19	TRNLTYIDPDALKEL	73	87	11	19	EIVETLGFNLTDTPM	73	87	11	19	AEIMETSIQAMKRKV	73	87	11
20	YIDPDALKELPLLKF	77	91	11	20	LGFNLTDTPMVEIQH	77	91	11	20	TSIQAMKRKVNLKTQ	77	91	11
21	ALKELPLLKFLGIFN	81	95	11	21	TDTPMVEIQHGFQHL	81	95	11	21	MKRKVNLKTQQSQHP	81	95	11
22	PLLKFLGIFNTGLKM	85	99	11	22	VEIQHGFQHLICSLN	85	99	11	22	NLKTQQSQHPTDALS	85	99	11
23	LGIFNTGLKMFPDLT	89	103	11	23	GFQHLICSLNFPKKE	89	103	11	23	QSQHPTDALSEDLLS	89	103	11
24	TGLKMFPDLTKVYST	93	107	11	24	ICSLNFPKKELELQI	93	107	11	24	TDALSEDLLSIIANM	93	107	11
25	FPDLTKVYSTDIFFI	97	111	11	25	FPKKELELQIGNALF	97	111	11	25	EDLLSIIANMSGCLP	97	111	11
26	KVYSTDIFFILEITD	101	115	11	26	LELQIGNALFIGKHL	101	115	11	26	IIANMSGCLPYMLPP	101	115	11
27	DIFFILEITDNPYMT	105	119	11	27	GNALFIGKHLKPLAK	105	119	11	27	SGCLPYMLPPKCPNT	105	119	11
28	LEITDNPYMTSIPVN	109	123	11	28	IGKHLKPLAKFLNDV	109	123	11	28	YMLPPKCPNTCLANK	109	123	11
29	NPYMTSIPVNAFQGL	113	127	11	29	KPLAKFLNDVKTLYE	113	127	11	29	KCPNTCLANKYRPIT	113	127	11
30	SIPVNAFQGLCNETL	117	131	11	30	FLNDVKTLYETEVFS	117	131	11	30	CLANKYRPITGACNN	117	131	11
31	AFQGLCNETLTLKLY	121	135	11	31	KTLYETEVFSTDFSN	121	135	11	31	YRPITGACNNRDHPR	121	135	11
32	CNETLTLKLYNNGFT	125	139	11	32	TEVFSTDFSNISAAK	125	139	11	32	GACNNRDHPRWGASN	125	139	11
33	TLKLYNNGFTSVQGY	129	143	11	33	TDFSNISAAKQEINS	129	143	11	33	RDHPRWGASNTALAR	129	143	11
34	NNGFTSVQGYAFNGT	133	147	11	34	ISAAKQEINSHVEMQ	133	147	11	34	WGASNTALARWLPPV	133	147	11
35	SVQGYAFNGTKLDAV	137	151	11	35	QEINSHVEMQTKGKV	137	151	11	35	TALARWLPPVYEDGF	137	151	11
36	AFNGTKLDAVYLNKN	141	155	11	36	HVEMQTKGKVVGLIQ	141	155	11	36	WLPPVYEDGFSQPRG	141	155	11
37	KLDAVYLNKNKYLTV	145	159	11	37	TKGKVVGLIQDLKPN	145	159	11	37	YEDGFSQPRGWNPGF	145	159	11
38	YLNKNKYLTVIDKDA	149	163	11	38	VGLIQDLKPNTIMVL	149	163	11	38	SQPRGWNPGFLYNGF	149	163	11
39	KYLTVIDKDAFGGVY	153	167	11	39	DLKPNTIMVLVNYIH	153	167	11	39	WNPGFLYNGFPLPPV	153	167	11
40	IDKDAFGGVYSGPSL	157	171	11	40	TIMVLVNYIHFKAQW	157	171	11	40	LYNGFPLPPVREVTR	157	171	11
41	FGGVYSGPSLLDVSQ	161	175	11	41	VNYIHFKAQWANPFD	161	175	11	41	PLPPVREVTRHVIQV	161	175	11
42	SGPSLLDVSQTSVTA	165	179	11	42	FKAQWANPFDPSKTE	165	179	11	42	REVTRHVIQVSNEVV	165	179	11
43	LDVSQTSVTALPSKG	169	183	11	43	ANPFDPSKTEDSSSF	169	183	11	43	HVIQVSNEVVTDDDR	169	183	11
44	TSVTALPSKGLEHLK	173	187	11	44	PSKTEDSSSFLIDKT	173	187	11	44	SNEVVTDDDRYSDLL	173	187	11
45	LPSKGLEHLKELIAR	177	191	11	45	DSSSFLIDKTTTVQV	177	191	11	45	TDDDRYSDLLMAWGQ	177	191	11
46	LEHLKELIARNTWTL	181	195	11	46	LIDKTTTVQVPMMHQ	181	195	11	46	YSDLLMAWGQYIDHD	181	195	11
47	ELIARNTWTLKKLPL	185	199	11	47	TTVQVPMMHQMEQYY	185	199	11	47	MAWGQYIDHDIAFTP	185	199	11
48	NTWTLKKLPLSLSFL	189	203	11	48	PMMHQMEQYYHLVDM	189	203	11	48	YIDHDIAFTPQSTSK	189	203	11
49	KKLPLSLSFLHLTRA	193	207	11	49	MEQYYHLVDMELNCT	193	207	11	49	IAFTPQSTSKAAFGG	193	207	11
50	SLSFLHLTRADLSYP	197	211	11	50	HLVDMELNCTVLQMD	197	211	11	50	QSTSKAAFGGGADCQ	197	211	11
51	HLTRADLSYPSHCCA	201	215	11	51	ELNCTVLQMDYSKNA	201	215	11	51	AAFGGGADCQMTCEN	201	215	11
52	DLSYPSHCCAFKNQK	205	219	11	52	VLQMDYSKNALALFV	205	219	11	52	GADCQMTCENQNPCF	205	219	11
53	SHCCAFKNQKKIRGI	209	223	11	53	YSKNALALFVLPKEG	209	223	11	53	MTCENQNPCFPIQLP	209	223	11
54	FKNQKKIRGILESLM	213	227	11	54	LALFVLPKEGQMESV	213	227	11	54	QNPCFPIQLPEEARP	213	227	11
55	KIRGILESLMCNESS	217	231	11	55	LPKEGQMESVEAAMS	217	231	11	55	PIQLPEEARPAAGTA	217	231	11
56	LESLMCNESSMQSLR	221	235	11	56	QMESVEAAMSSKTLK	221	235	11	56	EEARPAAGTACLPFY	221	235	11
57	CNESSMQSLRQRKSV	225	239	11	57	EAAMSSKTLKKWNRL	225	239	11	57	AAGTACLPFYRSSAA	225	239	11
58	MQSLRQRKSVNALNS	229	243	11	58	SKTLKKWNRLLQKGW	229	243	11	58	CLPFYRSSAACGTGD	229	243	11
59	QRKSVNALNSPLHQE	233	247	11	59	KWNRLLQKGWVDLFV	233	247	11	59	RSSAACGTGDQGALF	233	247	11
60	NALNSPLHQEYEENL	237	251	11	60	LQKGWVDLFVPKFSI	237	251	11	60	CGTGDQGALFGNLST	237	251	11
61	PLHQEYEENLGDSIV	241	255	11	61	VDLFVPKFSISATYD	241	255	11	61	QGALFGNLSTANPRQ	241	255	11
62	YEENLGDSIVGYKEK	245	259	11	62	PKFSISATYDLGATL	245	259	11	62	GNLSTANPRQQMNGL	245	259	11
63	GDSIVGYKEKSKFQD	249	263	11	63	SATYDLGATLLKMGI	249	263	11	63	ANPRQQMNGLTSFLD	249	263	11
64	GYKEKSKFQDTHNNA	253	267	11	64	LGATLLKMGIQHAYS	253	267	11	64	QMNGLTSFLDASTVY	253	267	11
65	SKFQDTHNNAHYYVF	257	271	11	65	LKMGIQHAYSENADF	257	271	11	65	TSFLDASTVYGSSPA	257	271	11

Supplementary Table S1

66	THNNAHYYVFFEEQE	261	275	11
67	HYYVFFEEQEDEIIG	265	279	11
68	FEEQEDEIIGFGQEL	269	283	11
69	DEIIGFGQELKNPQE	273	287	11
70	FGQELKNPQEETLQA	277	291	11
71	KNPQEETLQAFDSHY	281	295	11
72	ETLQAFDSHYDYTIC	285	299	11
73	FDSHYDYTICGDSED	289	303	11
74	DYTICGDSEDMVCTP	293	307	11
75	GDSEDMVCTPKSDEF	297	311	11
76	MVCTPKSDEFNPCED	301	315	11
77	KSDEFNPCEDIMG	305	317	11

66	QHAYSENADFSGLTE	261	275	11
67	ENADFSGLTEDNGLK	265	279	11
68	SGLTEDNGLKLSNAA	269	283	11
69	DNGLKLSNAAHKAVL	273	287	11
70	LSNAAHKAVLHIGEK	277	291	11
71	HKAVLHIGEKGTEAA	281	295	11
72	HIGEKGTEAAAVPEV	285	299	11
73	GTEAAAVPEVELSDQ	289	303	11
74	AVPEVELSDQPENTF	293	307	11
75	ELSDQPENTFLHPII	297	311	11
76	PENTFLHPIIQIDRS	301	315	11
77	LHPIIQIDRSFMLLI	305	319	11
78	QIDRSFMLLILERST	309	323	11
79	FMLLILERSTRSILF	313	327	11
80	LERSTRSILFLGKVV	317	331	11
81	RSILFLGKVVNPTEA	321	335	11
82	LGKVVNPTEA	325	334	11

66	ASTVYGSSPALERQL	261	275	11
67	GSSPALERQLRNWTS	265	279	11
68	LERQLRNWTSAEGLL	269	283	11
69	RNWTSAEGLLRVHAR	273	287	11
70	AEGLLRVHARLRDSG	277	291	11
71	RVHARLRDSGRAYLP	281	295	11
72	LRDSGRAYLPFVPPR	285	299	11
73	RAYLPFVPPRRPAAC	289	303	11
74	FVPPRRPAACAPEPG	293	307	11
75	RPAACAPEPGIPGET	297	311	11
76	APEPGIPGETRGPCF	301	315	11
77	IPGETRGPCFLAGDG	305	319	11
78	RGPCFLAGDGRASEV	309	323	11
79	LAGDGRASEVPSLTA	313	327	11
80	RASEVPSLTALHTLW	317	331	11
81	PSLTALHTLWLREHN	321	335	11
82	LHTLWLREHNRLAAA	325	339	11
83	LREHNRLAAALKALN	329	343	11
84	RLAAALKALNAHWSA	333	347	11
85	LKALNAHWSADAVYQ	337	351	11
86	AHWSADAVYQEARKV	341	355	11
87	DAVYQEARKVVGALH	345	359	11
88	EARKVVGALHQIITL	349	363	11
89	VGALHQIITLRDYIP	353	367	11
90	QIITLRDYIPRILGP	357	371	11
91	RDYIPRILGPEAFQQ	361	375	11
92	RILGPEAFQQYVGPY	365	379	11
93	EAFQQYVGPYEGYDS	369	383	11
94	YVGPYEGYDSTANPT	373	387	11
95	EGYDSTANPTVSNVF	377	391	11
96	TANPTVSNVFSTAAF	381	395	11
97	VSNVFSTAAFRFGHA	385	399	11
98	STAAFRFGHATIHPL	389	403	11
99	RFGHATIHPLVRRLD	393	407	11
100	TIHPLVRRLDASFQE	397	411	11
101	VRRLDASFQEHPDLP	401	415	11
102	ASFQEHPDLPGLWLH	405	419	11
103	HPDLPGLWLHQAFFS	409	423	11
104	GLWLHQAFFSPWTLL	413	427	11
105	QAFFSPWTLLRGGYN	417	431	11
106	PWTLLRGGYNEWREF	421	435	11
107	RGGYNEWREFCGLPR	425	439	11
108	EWREFCGLPRLETPA	429	443	11
109	CGLPRLETPADLSTA	433	447	11
110	LETPADLSTAIASRS	437	451	11
111	DLSTAIASRSVADKI	441	455	11
112	IASRSVADKILDLYK	445	459	11
113	VADKILDLYKHPDNI	449	463	11
114	LDLYKHPDNIDVWLG	453	467	11
115	HPDNIDVWLGGLAEN	457	471	11
116	DVWLGGLAENFLPRA	461	475	11
117	GLAENFLPRARTGPL	465	479	11
118	FLPRARTGPLFACLI	469	483	11
119	RTGPLFACLIGKQMK	473	487	11
120	FACLIGKQMKALRDG	477	491	11
121	GKQMKALRDGDWFWW	481	495	11
122	ALRDGDWFWWENSHV	485	499	11
123	DWFWWENSHVFTDAQ	489	503	11
124	ENSHVFTDAQRRELE	493	507	11
125	FTDAQRRELEKHSLS	497	511	11
126	RRELEKHSLSRVICD	501	515	11
127	KHSLSRVICDNTGLT	505	519	11
128	RVICDNTGLTRVPMD	509	523	11
129	NTGLTRVPMDAFQVG	513	527	11
130	RVPMDAFQVGKFPED	517	531	11
131	AFQVGKFPEDFESCD	521	535	11
132	KFPEDFESCDSIPGM	525	539	11

133	FESCDSIPGMNLEAW	529	543	11
134	SIPGMNLEAWRETFP	533	547	11
135	NLEAWRETFPQDDKC	537	551	11
136	RETFPQDDKCGFPES	541	555	11
137	QDDKCGFPESVENGD	545	559	11
138	GFPESVENGDFVHCE	549	563	11
139	VENGDFVHCEESGRR	553	567	11
140	FVHCEESGRRVLVYS	557	571	11
141	ESGRRVLVYSCRHGY	561	575	11
142	VLVYSCRHGYELQGR	565	579	11
143	CRHGYELQGREQLTC	569	583	11
144	ELQGREQLTCTQEGW	573	587	11
145	EQLTCTQEGWDFQPP	577	591	11
146	TQEGWDFQPPLCKDV	581	595	11
147	DFQPPLCKDVNECAD	585	599	11
148	LCKDVNECADGAHPP	589	603	11
149	NECADGAHPPCHASA	593	607	11
150	GAHPPCHASARCRNT	597	611	11
151	CHASARCRNTKGGFQ	601	615	11
152	RCRNTKGGFQCLCAD	605	619	11
153	KGGFQCLCADPYELG	609	623	11
154	CLCADPYELGDDGRT	613	627	11
155	PYELGDDGRTCVDSG	617	631	11
156	DDGRTCVDSGRLPRA	621	635	11
157	CVDSGRLPRATWISM	625	639	11
158	RLPRATWISMSLAAL	629	643	11
159	TWISMSLAALLIGGF	633	647	11
160	SLAALLIGGFAGLTS	637	651	11
161	LIGGFAGLTSTVICR	641	655	11
162	AGLTSTVICRWTRTG	645	659	11
163	TVICRWTRTGTKSTL	649	663	11
164	WTRTGTKSTLPISET	653	667	11
165	TKSTLPISETGGGTP	657	671	11
166	PISETGGGTPELRCG	661	675	11
167	GGGTPELRCGKHQAV	665	679	11
168	ELRCGKHQAVGTSPQ	669	683	11
169	KHQAVGTSPQRAAAQ	673	687	11
170	GTSPQRAAAQDSEQE	677	691	11
171	RAAAQDSEQESAGME	681	695	11
172	DSEQESAGMEGRDTH	685	699	11
173	SAGMEGRDTHRLPRA	689	703	11
174	GRDTHRLPRAL	693	704	11

Supplementary Table S2A. Gene expression changes in total B cells of PBMC in response to overlapping peptides of TSHR, Tg, and TPO antigens compared to control

Total B cells							
	Negative log2FC(<-1)				Positive log2FC(>1)		
gene	TSHR	Tg	TPO	gene	TSHR	Tg	TPO
CCL5	-2.738	-2.706	-2.568	CD22	0.982	1.020	1.104
CD247	-1.116	-1.119	-1.128	CD37	1.198	1.191	1.230
CD3E	-1.389	-1.326	-1.314	CD74	2.078	2.177	2.280
CD8A	-1.124	-1.026	-0.985	CD79A	2.246	2.273	2.330
CST7	-1.646	-1.689	-1.564	CXCR5	0.903	0.938	1.123
CTSW	-1.531	-1.561	-1.508	HLA-DMA	1.285	1.529	1.620
CXCL8	-1.476	-1.020	-1.132	HLA-DPA1	1.435	1.495	1.637
FYN	-1.263	-1.302	-1.306	HLA-DQB1	1.447	1.510	1.606
GNLY	-2.600	-2.739	-2.575	HLA-DRA	2.932	3.045	3.134
GZMA	-1.149	-1.143	-1.118	IGHA1-secreted	2.137	2.942	2.667
GZMB	-1.675	-1.734	-1.682	IGHD-membrane	1.293	1.338	1.188
GZMH	-1.103	-1.132	-1.100	IGHG1-secreted	1.254	2.090	2.014
IL2RB	-1.782	-1.821	-1.734	IGHG2-secreted	1.462	2.564	2.453
IL32	-2.310	-2.241	-2.198	IGHM-membrane	1.658	1.606	1.602
ITGB2	-1.326	-1.312	-1.159	IGHM-secreted	1.183	1.376	1.289
KLRB1	-1.187	-1.136	-1.070	IGKC	2.560	3.046	3.113
LCK	-0.996	-1.017	-0.969	IGLC3	2.541	2.495	2.399
NKG7	-2.891	-2.847	-2.748	IRF8	0.953	1.038	1.355
PRF1	-1.866	-1.884	-1.824	JCHAIN	1.323	1.740	1.478
SELPLG	-1.190	-1.029	-1.016	MS4A1	1.606	1.642	1.782
TRAC	-0.916	-1.022	-1.010	QPCT	0.686	1.477	1.299
TRBC2	-1.262	-1.134	-1.169	YBX3	1.248	1.349	0.945

Abbreviations: PBMC: peripheral blood mononuclear cell; FC, fold change; TSHR: thyroid-stimulating hormone receptor; Tg, thyroglobulin; TPO, thyroid peroxidase.

Genes with |log2FC|>1 compared to control are shown. Total B cells are the combined of naive and memory B cells.

Supplementary Table S2B. Gene expression changes in naive B cells of PBMC in response to overlapping peptides of TSHR, Tg, and TPO antigens compared to control

Naive B cells							
	Negative log2FC(<-1)				Positive log2FC(>1)		
gene	TSHR	Tg	TPO	gene	TSHR	Tg	TPO
CCL5	-2.856	-2.760(n.s.)	-2.719	CD69	0.976	1.031	0.950
CD2	-1.023	-0.991	-0.993	CD74	1.631	1.927	2.080
CD247	-1.177	-1.102	-1.183	CD79A	1.854	1.990	2.094
CD3E	-1.475	-1.380	-1.395	CXCR5	0.821	0.872	1.028
CD8A	-1.156	-1.094	-1.105	HLA-DMA	1.056	1.376	1.522
CST7	-1.691	-1.689	-1.621	HLA-DPA1	0.849	1.188	1.370
CTSW	-1.528	-1.564	-1.541	HLA-DQB1	0.891	1.204	1.351
FYN	-1.532	-1.413	-1.501	HLA-DRA	2.826	3.014	3.088
GAPDH	-1.204	-0.611	-0.627	IGHA1-secreted	2.373	2.562	2.645
GNLY	-2.647	-2.701	-2.760	IGHG1-secreted	0.392	1.607	1.834
GZMA	-1.125	-1.149	-1.133	IGHG2-secreted	0.375	2.021	2.262
GZMB	-1.848	-1.806	-1.748	IGHM-membrane	0.093	0.915	1.087
GZMH	-1.120	-1.132	-1.114	IGKC	2.107	2.416	2.859
IFITM3	-1.008	-0.884	-0.782	IGLC3	2.578	2.367	2.334
IL1B	-0.733	-0.656	-1.131	IRF8	1.010	0.949	1.382
IL2RB	-1.808	-1.875	-1.873	JCHAIN	1.440	1.328	1.329
IL32	-2.317	-2.254	-2.274	MS4A1	1.073	1.242	1.511
IL7R	-1.045	-1.023	-1.114	QPCT	0.672(n.s.)	1.598	1.353
ITGB2	-1.610	-1.467	-1.342	YBX3	1.398	1.487	1.125
KLRB1	-1.218	-1.130	-1.101				
KLRK1	-1.096	-1.008	-0.980				
LCK	-1.026	-1.061	-0.999				
LGALS1	-1.055	-0.819	-0.660				
NKG7	-2.929	-2.821(n.s.)	-2.847(n.s.)				
PRF1	-1.861	-1.886	-1.865				
PTPRC	-1.252	-0.842	-0.807				
SELPLG	-1.217	-1.087	-1.092				
TRAC	-0.961	-1.065	-1.145				
TRBC2	-1.481	-1.328	-1.353				

Abbreviations: PBMC: peripheral blood mononuclear cell; FC, fold change; TSHR: thyroid-stimulating hormone receptor; Tg, thyroglobulin; TPO, thyroid peroxidase.

Genes with |log2FC|>1 compared to control are shown.

Supplementary Table S2C. Gene expression changes in memory B cells of PBMC in response to overlapping peptides of TSHR, Tg, and TPO antigens compared to control

Memory B cells							
	Negative log2FC(<-1)				Positive log2FC(>1)		
gene	TSHR	Tg	TPO	gene	TSHR	Tg	TPO
CCL5	-2.479	-2.589	-2.284(n.s.)	CD1C	1.154	0.972	0.830
CD247	-0.974	-1.161	-1.016	CD22	1.775	1.631	1.600
CD3E	-1.193	-1.206	-1.151	CD24	1.093	0.941	0.861
CD8A	-1.047	-0.877(n.s)	-0.755	CD37	1.957	1.704	1.657
CST7	-1.539	-1.691	-1.449(n.s.)	CD40	0.828	1.202	1.062
CTSW	-1.538	-1.552	-1.439	CD52	1.324	1.091	1.105
CXCL8	0.371(n.s.)	-1.300	-1.214	CD74	2.815	2.637	2.638
FCGR3A	-1.104	-0.985	-1.067	CD79A	2.920	2.777	2.740
FYN	-0.751	-1.069	-0.956	CD79B	1.833	1.654	1.513
GNLY	-2.488	-2.833	-2.240	CXCR4	1.247	0.892	0.905
GZMA	-1.209	-1.130	-1.085	CXCR5	1.089	1.083	1.311
GZMB	-1.314	-1.579(n.s.)	-1.549	EGR1	0.361	0.232	1.003
GZMH	-1.062	-1.133(n.s.)	-1.070	HLA-DMA	1.735	1.834	1.814
IL2RB	-1.719	-1.701	-1.471	HLA-DPA1	2.311	2.032	2.088
IL32	-2.292	-2.211	-2.044	HLA-DQB1	2.294	2.045	2.043
ITGB2	-0.794	-1.002	-0.826	HLA-DRA	3.167	3.116	3.228
KLRB1	-1.111	-1.151	-1.004	IGHA1-secreted	1.292	3.568	2.713
NKG7	-2.802	-2.907	-2.552	IGHD-membrane	2.116	1.925	1.660
PRF1	-1.879(n.s.)	-1.878(n.s.)	-1.738(n.s.)	IGHG1-secreted	2.341	2.829	2.343
SELPLG	-1.123	-0.902	-0.865	IGHG2-secreted	2.678	3.361	2.797
				IGHM-membrane	2.999	2.529	2.327
				IGHM-secreted	2.401	2.432	2.110
				IGKC	3.303	3.920	3.547
				IGLC3	2.445	2.758	2.530
				IRF8	0.801	1.229	1.296
				JCHAIN	0.980	2.404	1.757
				MS4A1	2.432	2.292	2.239
				PAX5	1.121	1.005	0.967
				POU2AF1	1.238	1.395	1.342
				QPCT	0.720	1.142	1.173
				TCL1A	1.396	1.101	1.090
				VPREB3	1.221	0.984	0.958

Abbreviations: PBMC: peripheral blood mononuclear cell; FC, fold change; TSHR: thyroid-stimulating hormone receptor; Tg, thyroglobulin; TPO, thyroid peroxidase.

Genes with |log2FC|>1 compared to control are shown.
